# Fibrillation/defibrillation of myoglobin decorated with gold nanoparticles probed through nanometal surface energy transfer mechanism

**DOI:** 10.1039/d6ra02822e

**Published:** 2026-07-02

**Authors:** Shalini Dyagala, Chien-Hsiang Chang, Subit Kumar Saha

**Affiliations:** a Department of Chemistry, Birla Institute of Technology and Science Pilani, Hyderabad Campus Hyderabad Telangana 500078 India sksaha@hyderabad.bits-pilani.ac.in sksaha@pilani.bits-pilani.ac.in +91-40-66303643; b Department of Chemical Engineering, National Cheng Kung University No. 1, University Rd., East Dist. Tainan 70101 Taiwan

## Abstract

Amyloid fibrillation and protein aggregation are closely associated with several pathological and structural transformations, making it important to understand their conformational heterogeneity and microenvironmental properties. In the present study, structural transitions of equine skeletal myoglobin (EMb) conjugated with gold nanoparticles (AuNPs) from native to amorphous aggregates, cross-β amyloid fibrils, and partially refolded states induced by sodium dodecyl sulfate (SDS) were investigated through nanometal surface energy transfer (NSET)-based photophysical approaches. Intrinsic aromatic amino acid residues, along with Coumarin-153 (C153) and Rhodamine-6G (Rh6G) as interior- and surface-sensitive fluorescent probes, respectively, were employed to probe the microenvironmental heterogeneity and energy-transfer behavior of different conformational states. Intrinsic fluorescence studies demonstrated state-dependent quenching predominantly governed by non-radiative decay pathways, where amorphous aggregates exhibited the highest quenching efficiency due to enhanced structural disorder and greater fluorophore accessibility to AuNPs. Fluorescence lifetime measurements of C153 revealed an open interior matrix in amorphous aggregates that promoted maximum energy-transfer efficiency, whereas the ordered cross-β-sheet architecture of amyloid fibrils shielded the probe within the fibrillar core, resulting in reduced energy transfer and enhanced excited-state lifetime. Time-resolved anisotropy studies further indicated that, despite their open structure, amorphous aggregates possessed a comparatively rigid interior microenvironment, while amyloid fibrils exhibited weaker immobilization within the fibrillar core but a relatively rigid exterior environment. Both amorphous aggregates and amyloid fibrils were found to possess less flexible interior and exterior environments than native bioconjugates. Furthermore, the photophysical behavior of intrinsic and extrinsic fluorophores supported the defibrillation of protein structures at SDS concentrations above the critical micelle concentration. Overall, this study demonstrates the effectiveness of NSET-based photophysical techniques for monitoring protein aggregation, amyloid fibrillation, microenvironmental heterogeneity, and defibrillation processes in bioconjugates.

## Abstract 2

Surfactant-induced structural modulation of myoglobin-AuNP bioconjugates through formation of amorphous aggregates, ordered amyloid fibrils, and partially refolded protein probed using NSET from intrinsic/extrinsic fluorescent donors to AuNP.

## Graphical abstract

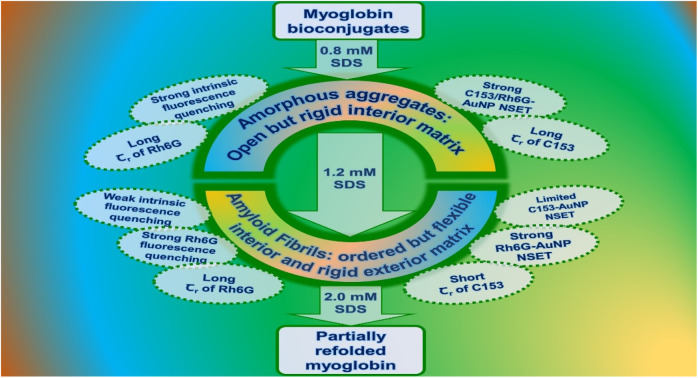

## 1 Introduction

Protein aggregation and amyloid fibril formation are fundamental phenomena underlying numerous neurodegenerative and systemic disorders, as well as functional biological assemblies.^1,2^ Myoglobin, a small globular heme protein, has served as a valuable model system for investigating protein folding, unfolding, and aggregation due to its well-defined tertiary structure and sensitivity to chemical perturbers such as surfactants.^3,4^ Sodium dodecyl sulfate (SDS), in particular, induces a series of concentration-dependent structural transitions in myoglobin, progressing from native folded states to amorphous aggregates, ordered cross-β secondary structure of amyloid fibrils, and ultimately resolubilized conformations above the critical micelle concentration.^5^ SDS is often used as a model of an anionic surfactant because its amphiphilic structure (hydrophobic tail and anionic head) mimics biological membranes, lipid bilayers, and endogenous amphiphilic molecules such as phospholipids and fatty acids.^6^ This allows for controlled induction of protein misfolding, interactions at membrane interfaces, and destabilization of native protein structures through electrostatic interactions with positively charged residues and hydrophobic insertion.^7^ Even within the body, membrane disruption occurs during cellular stress, apoptosis, or lysosomal leakage, exposing proteins to anionic lipids such as bis(monoacylglycero)phosphate, phosphatidylserine, and free fatty acids. These lipids similarly trigger protein misfolding and aggregation, resembling the effects of SDS.^8^ Tofani and co-workers^9^ have shown that SDS binds to myoglobin in the monomeric form, suggesting specific binding. Feis *et al.*^10^ have reported the effect of surfactant concentration, *i.e.*, various aggregation states, on the modes of interaction. Despite extensive studies on various states of aggregation, fibrillation, and defibrillation of proteins, far less is known about how these structures are altered and what microenvironmental changes occur at the protein's interior and exterior during structural transformations, though they are important to study as far as their relation with neurodegenerative and systemic disorders is concerned.

Gold nanoparticles (AuNPs) are widely employed as model nanomaterials due to their well-defined optical properties, biocompatibility, and strong interactions with proteins. Protein adsorption onto AuNP surfaces leads to the formation of a dynamic protein corona, which can profoundly influence protein conformation, aggregation pathways, and photophysical behaviour. AuNPs are being explored for therapeutic applications and medical diagnosis.^11–13^ As they are safe for the human body, they are attractive for basic research on their interactions with biomaterials and for physicochemical studies of model systems.^14,15^ Gold is preferred over other metals in this field of research because AuNPs exhibit minimal interactions with other chemicals present, such as polymers or surfactants.^14^ Notably, AuNPs act as efficient energy acceptors, enabling distance-dependent fluorescence quenching *via* nanomaterial surface energy transfer (NSET), making them powerful probes for nanoscale structural changes.^16–18^ Compared with fluorescence resonance energy transfer (FRET), the NSET mechanism has the advantage of not being limited by the distance between the donor (D) and acceptor (A), unlike FRET.^19–22^ While there is a limit of D–A distance for up to 90 A° for the FRET method to be applicable, that can be overcome in the NSET method, which is based on the principle of interactions between the electromagnetic field of the dipole of D and the delocalized electrons present in the conduction band of A.^23,24^ While AuNP-induced modulation of native protein fluorescence has been widely reported, systematic studies correlating nanoparticle-mediated energy transfer with well-defined protein aggregation states remain limited. Bioconjugates formed through protein-nanoparticle interactions integrate biological functionality with the unique physicochemical properties of nanomaterials, thereby enhancing protein stability and reducing amyloid-associated cytotoxicity.^25^ These interactions can stabilize proteins against fibrillation by surface passivation and modulate folding pathways through interfacial effects at the nanoparticle surface.^9^ Additionally, such systems enable quantitative analysis of conformational dynamics *via* distance-dependent energy transfer mechanisms to assess structural changes and unfolding distances. Beyond fundamental biophysical insights, these bioconjugates hold significant potential for nanomedicine applications, including biosensing, targeted drug delivery, and therapeutic strategies to mitigate protein aggregation diseases.^25^

In this context, the present work aims to bridge protein aggregation science with nanoparticle photophysics by examining SDS-induced structural transitions in equine skeletal myoglobin-gold nanoparticle (EMb-AuNP) bioconjugates. The EMb is a small globular protein having 153 amino acids.^26^ It has been chosen for bioconjugation because it lacks cysteine residues, thereby preventing covalent bonding to the AuNP surface. Thus, the EMb–AuNPs complex forms *via* hydrophobic and electrostatic interactions, yielding a bioconjugate lacking specific binding sites.^26^ EMb has been used for a wide range of studies, from crystallographic analysis^27^ to spectroscopic applications across various forms, including free and ligated proteins and chemically distinct environments.^14,28–33^ That is why EMb is considered a protein of simple and versatile nature. Sevilla *et al.*^14^ have shown that EMb protein coating on AuNPs surfaces prevents the nanoparticles from aggregating and/or from optical coupling.

By combining turbidity measurements, intrinsic aromatic amino acid fluorescence,^5^ thioflavin-T assays,^5,34^ and electron microscopy, we establish a nanoparticle-modified aggregation landscape encompassing native, amorphous aggregates, cross-β secondary structure of amyloid fibrils, and partially refolded states. Importantly, we have integrated steady-state and time-resolved fluorescence spectroscopy, fluorescence anisotropy measurements, and analyses of radiative and non-radiative decay rate constants to quantify how AuNP proximity modulates energy dissipation across these states.

**Scheme 1:**
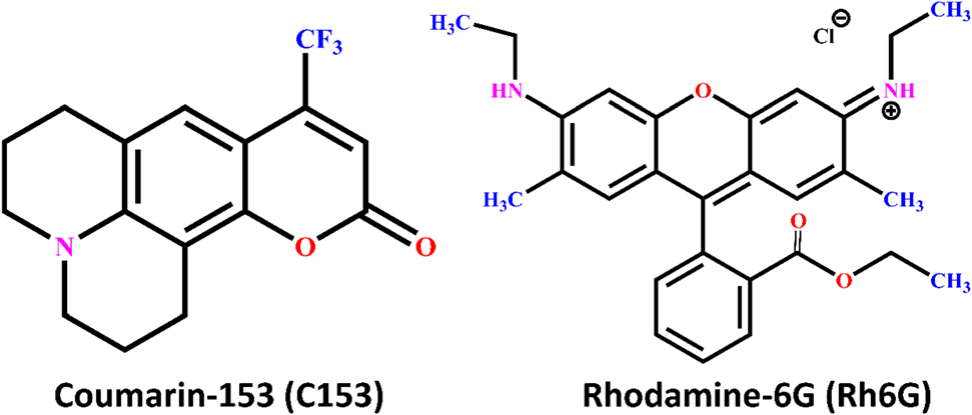
Chemical structures of C153 and Rh6G.

To further resolve spatial heterogeneity within aggregated structures, two complementary fluorescent probes, Coumarin-153 (C153) and Rhodamine-6G (Rh6G) [Scheme 1], were employed. C153 serves as a reporter probe for the protein's interior, whereas Rh6G preferentially reports on surface and interfacial regions.^16,35,36^ By correlating systematic studies with location-specific dyes' fluorescence lifetimes, fluorescence anisotropy decays, and energy-transfer efficiencies as a function of protein's structural changes, results provide a unified mechanistic picture of how disorder, fibrillar ordering, and nanoparticle interfaces collectively govern photophysical responses. Studies report overall information on microenvironmental changes within and around the protein during its structural transformation.

## 2 Experimental Section

Comprehensive information on the materials and methods employed is provided in Section S1 of the SI. Information regarding the materials is provided in Section S1.1. Details on protein sample preparation and the various measurement methods and instruments employed are provided in Section S1.2. The methods used to calculate FRET and NSET parameters are described in Section S1.3. A few figures and tables related to the results are also available in the SI.

## 3 Results and discussion

### 3.1 Synthesis of EMb-AuNP bioconjugates

EMb-AuNP bioconjugates were synthesized by mixing 0.1 µM EMb (16.9 mg) in 9.5 mL of 20 mM PBS buffer (pH 7.4) with 0.5 mL of HAuCl_4_ solution (∼10 mM). After stirring for 5 min, 100 µL of freshly prepared ice-cold NaBH_4_ aqueous solution (∼0.001 g mL^−1^) was added under continuous stirring. The reaction mixture was stirred for 2 hours and subsequently allowed to settle. The precipitate was centrifuged at 2500 rpm and washed three times to remove unbound EMb. The concentration of the as-prepared AuNPs stock solution was 0.124 µM, which was further diluted to a working concentration of 0.0124 µM as required. The colloidal solutions were then refrigerated carefully at 4 °C until further use. It was assumed that the reduction process resulted in complete conversion of Au^3+^ to Au^0^.^18,37^

The isoelectric point (pI) of EMb in the bioconjugate system, determined from conductivity measurements, was 6.5 as given in Fig. S1. At pH values approximately two units below the pI, the protein carries a net positive charge due to protonation of basic residues. Accordingly, experiments were performed at pH 4.5 to investigate the interaction between positively charged EMb-AuNP bioconjugates and negatively charged sodium dodecyl sulfate (SDS).

### 3.2 Characterization of EMb-AuNP bioconjugates

#### 3.2.1 UV-Vis absorption spectral characterization

**Fig. 1:**
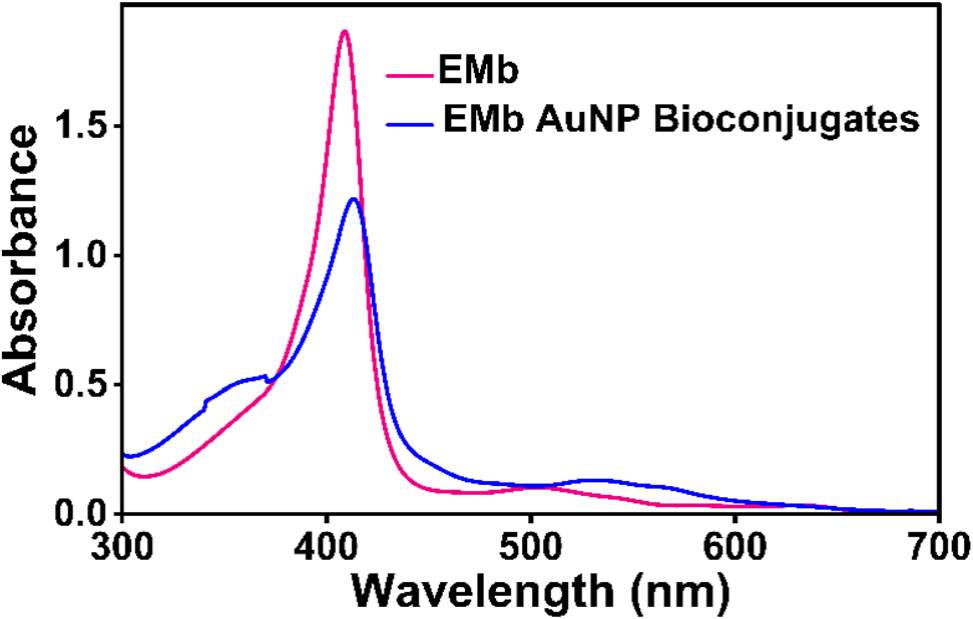
UV-visible absorption spectra of native EMb and EMb-AuNP bioconjugates in an acetate buffer of pH = 4.5. [EMb] = [EMb bioconjugate] = 10.00 µM, [AuNPs] = 0.0124 µM.

The details of UV-vis absorption spectral measurements are given in Section S1.2.2. The UV-vis absorption spectrum of native equine skeletal myoglobin (EMb) showed a strong Soret band at ∼409 nm, characteristic of the intact heme environment in the folded protein (Fig. 1).^9,38^ According to reports, AuNPs exhibit a distinct surface plasmon resonance (SPR) peak at ∼520 nm.^39,40^ Upon the formation of bioconjugates, this SPR band underwent slight broadening accompanied by a small red-shift, indicating changes in the local refractive index at the nanoparticle–protein interface, as shown in Fig. 1. A modest decrease in Soret band intensity was also observed, suggesting adsorption of EMb onto the AuNP surface and partial shielding of the heme environment. These spectral features collectively confirmed the formation of EMb-AuNP bioconjugates.

#### 3.2.2 High resolution transmission electron microscopy (HR-TEM)

**Fig. 2:**
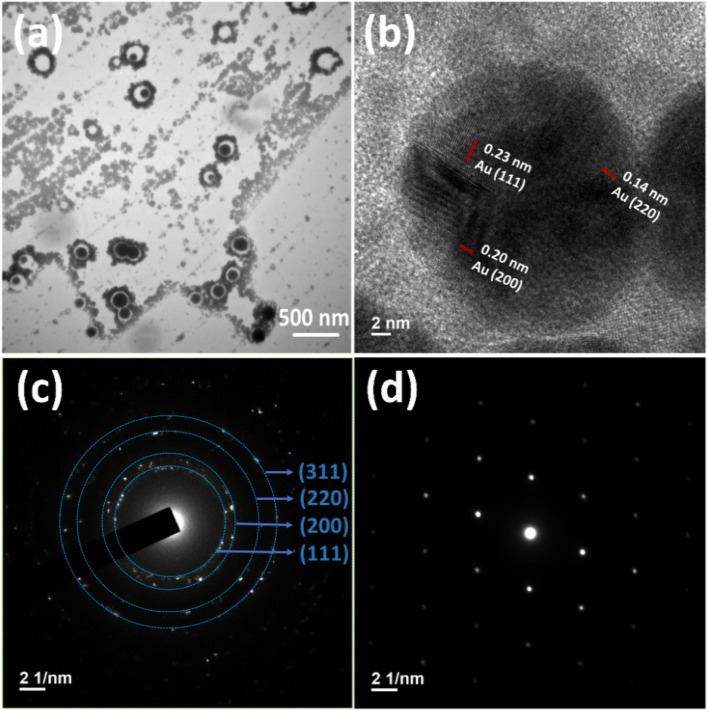
HR-TEM images of EMb bioconjugates (a), lattice fringes (b), SAED (c), and NBED (d). [EMb bioconjugate] = 10.00 µM, [AuNPs] = 0.0124 µM.

The detailed measurement methods are described in Section S1.2.3. HR-TEM analysis (Fig. 2a–d) of EMb-AuNP bioconjugates reveals predominantly spherical nanoparticles with core diameters of ∼20–25 nm surrounded by a thin, low-contrast protein corona (Fig. 2a), confirming successful EMb conjugation without large-scale aggregation. *In situ* synthesis of AuNPs within myoglobin bioconjugates yielded well-crystalline fcc gold, as evidenced by HRTEM lattice fringes corresponding to Au (111), (200), and (220) planes (Fig. 2b). Selected Area Electron Diffraction (SAED) patterns (Fig. 2c) exhibit concentric rings, *i.e.*, polycrystallinity consistent with lattice fringes data. The Nano Beam Electron Diffraction (NBED) pattern (Fig. 2d) of a single nanoparticle exhibits discrete diffraction spots with well-defined *d*-spacings, confirming its single-crystalline character and verifying high crystallinity suitable for bioconjugate applications, with the protein corona providing colloidal stability.

#### 3.2.3 X-ray photoelectron spectroscopy (XPS) and powder X-ray diffraction (PXRD)

**Fig. 3:**
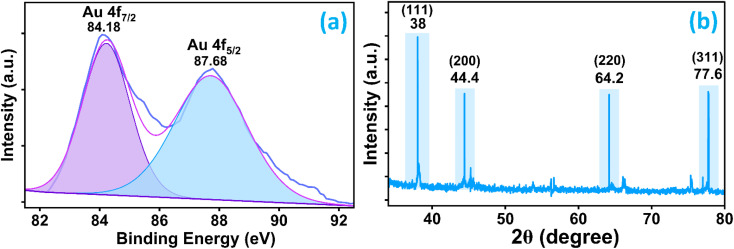
(a) XPS spectra and (b) PXRD patterns of *in situ* synthesized AuNPs of EMb bioconjugates. [EMb bioconjugate] = 10.00 µM, [AuNPs] = 0.0124 µM.

Instruments and sample preparation details are available in Section S1.2.4. High-resolution XPS analysis confirmed the chemical composition and oxidation states of *in situ* synthesized AuNPs within EMb bioconjugates (Fig. 3a). Spectral deconvolution employed Shirley-type background correction for precise peak fitting. The Au 4f region revealed characteristic doublets at 84.18 eV (Au 4f_7/2_) and 87.68 eV (Au 4f_5/2_), diagnostic of metallic (Au^0^). The 3.5 eV spin–orbit splitting further verifies complete reduction to elemental gold within the protein matrix.

PXRD analysis (Fig. 3b) corroborated AuNP formation, displaying prominent Bragg reflections at 2*θ* = 38.0°, 44.4°, 64.2°, and 77.6°, indexing to the (111), (200), (220), and (311) planes of face-centered cubic (fcc) Au, respectively. The sharp, well-defined peaks evidence high crystallinity of the AuNPs, consistent with HR-TEM observations of single-crystalline domains.

#### 3.2.4 Dynamic light scattering (DLS) and zeta potential measurements

Details of measurement methods are given in Section S1.2.5. DLS analysis of the *in situ* synthesized EMb-AuNP bioconjugates, as shown in Fig. S2(a), revealed a hydrodynamic diameter of 36 ± 0.35 nm with a polydispersity index (PDI) of 0.35, confirming the formation of stable, protein-coated nanoparticles in aqueous solution with minimal aggregation. The ∼36 nm hydrodynamic diameter is significantly larger than the ∼10–20 nm TEM core size, as expected for hydrated protein layers (5–10 nm thick) surrounding individual AuNPs, further validating the robust EMb coating observed in HR-TEM.

Zeta potential measurements yielded a surface charge of +29 mV (Fig. S2(b)), ensuring excellent colloidal stability consistent with successful bioconjugation. It almost matches the reported values for hemoglobin-AuNPs bioconjugates; the zeta potential of +35 mV was observed for the bioconjugates, whereas citrate-capped AuNPs showed a value of −50 mV.^41,42^

#### 3.3 SDS-induced structural transitions in EMb-AuNP bioconjugates

After establishing the successful preparation and characterization of EMb-AuNP bioconjugates, we next examined how SDS modulates their structural state. SDS is known to induce a series of conformational transitions in myoglobin, progressing from native protein to amorphous aggregates and finally to ordered amyloid-like fibrils with a cross-β secondary structure, depending on concentration. However, these transitions have never been evaluated for EMb-AuNP bioconjugates, where the AuNP surface may alter unfolding, aggregation, and the nature of intermediate states. To systematically probe these changes, we performed turbidity measurements, intrinsic fluorescence spectroscopy, ThT binding assays, far-UV CD spectroscopy, and HR-TEM imaging.

#### 3.3.1 Turbidity-based evaluation of SDS-induced structural transitions in EMb-AuNP bioconjugates

**Fig. 4:**
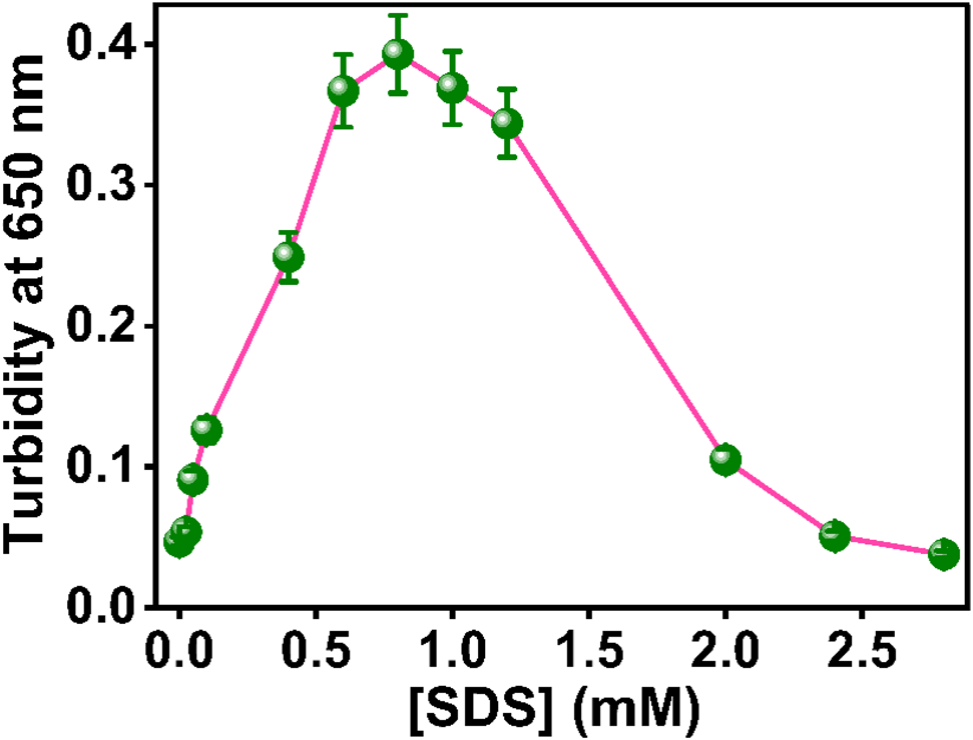
Turbidity of EMb-AuNP bioconjugate samples treated with different concentrations of SDS in the acetate buffer of pH = 4.5. Turbidity was measured by absorbance at 650 nm. [EMb bioconjugate] = 10.00 µM, [AuNPs] = 0.0124 µM.

Details of instrumental methods and sample preparation are described in Sections S.1.2.2 and S1.2.6, respectively. SDS-driven changes in EMb-AuNP bioconjugates were tracked using turbidity, measuring absorbance at 650 nm. Turbidity measures the clearance or cloudiness of liquids. Proteins treated with chemicals or incubated under stress conditions undergo structural changes and altered solubility, leading to changes in opacity. EMb-AuNP bioconjugates were soluble at pH 4.5, and no change in turbidity was observed. But turbidity changes were observed in a concentration-dependent manner in the presence of SDS. From Fig. 4, we can clearly see a sharp increase in absorbance above ∼0.1 mM SDS, indicating the onset of aggregate formation. The turbidity reached a maximum around ∼0.8 mM SDS, consistent with the formation of large, light-scattering protein SDS clusters. At higher SDS concentrations (>1.8 mM), turbidity decreased progressively, suggesting protein disaggregation and subsequent solubilization. The dual behaviour of SDS was due to its existence in soluble monomeric and micellar oligomeric states. The CMC of SDS in the presence of 10 µM of EMb–AuNPs bioconjugate with 0.0124 µM of AuNPs present in it is determined by conductivity measurement (Fig. S3) and is found to be 1.8 mM. Thus, SDS molecules exist in a monomeric form at a low concentration range of pre-micellar state (<1.8 mM) and in a micellar state at a higher concentration range (>1.8 mM). At a monomeric state of SDS molecules, aggregations occur as a result of electrostatic interactions between the negatively charged SDS and positively charged protein bioconjugate residues, and also hydrophobic interactions between the hydrocarbon tail of SDS and hydrophobic domains of protein bioconjugate. As protein-solvent contacts are perturbed by charge neutralisation, hydrophobic residues of protein bioconjugates are exposed, leading to aggregation. However, at SDS concentrations above its CMC, protein bioconjugate solubilization occurs because SDS molecules leave the aggregate and form micelles in solution.^5,43,44^ The upward shift of the turbidity maximum from 0.4 mM (free E-Mb)^5^ to ∼0.8 mM in the bioconjugates indicates that AuNP binding alters the balance between SDS-driven protein–protein interactions and surfactant-mediated solubilization.

#### 3.3.2 Intrinsic fluorescence measurements

Details of the steady-state spectrofluorimetric measurements are available in Section S1.2.2. Intrinsic fluorescence measurements of aromatic amino acid residues (*λ*_ex_ = 280 nm) of EMb-AuNP bioconjugates with increasing SDS concentrations were used to monitor tertiary structure perturbations of a protein. Detailed measurement methods of intrinsic fluorescence were taken from elsewhere^45,46^ and given in SI S1.2.7. As shown in Fig. S4, the fluorescence maxima of native EMb (without AuNPs) and EMb bioconjugate (both without SDS treatment) were observed at 330 and 335 nm, respectively. Fluorescence in bioconjugates is quenched by AuNPs. The 5.0 nm red-shift is due to mild exposure to the solvent environment. Upon addition of 0.1 mM SDS, the fluorescence intensity increases because two tryptophan residues (Trp-7 and Trp-14 on helix A) move away from the heme, reducing energy transfer between them.^9^ However, no substantial change in wavelength is noted in the case of bioconjugates. With further increasing SDS concentration, fluorescence is quenched and shows a red shift, as shown in Fig. S4, up to 1.2 mM SDS. It is due to the exposure of aromatic amino acid residues to AuNPs and the solvent environment. At higher SDS concentrations (above 1.8 mM), fluorescence intensity increases and shows a blue shift as the protein matrix is shielded during refolding. When BSA was treated with SDS, a similar effect was observed: a shift in the wavelength maximum was observed, indicating that the fluorophore (aromatic amino acids) had moved to a more hydrophobic environment.^47^

EMb-AuNP bioconjugates display a distinct conformational response due to strong nanoparticle protein interactions. Intrinsic fluorescence decreased up to 1.2 mM SDS and exhibited a red shift, which we attribute to SDS-induced disruption of the protein corona and enhanced NSET quenching between EMb aromatic amino acid residues and AuNPs, together with exposure of those residues to a more polar environment, indicating the formation of aggregates and ordered, amyloid-like assemblies around the nanoparticle surface. At higher SDS concentrations, the fluorescence intensity recovered slightly, indicating that the protein transitions back to a more native-like folded state. At this stage, SDS exists predominantly near and above its critical micellar concentration, supporting the resolubilization of protein assemblies and the recovery of tertiary structure, a trend that is retained even in nanoparticle-bound myoglobin systems.

#### 3.3.3 Thioflavin-T (ThT) binding assay

**Fig. 5:**
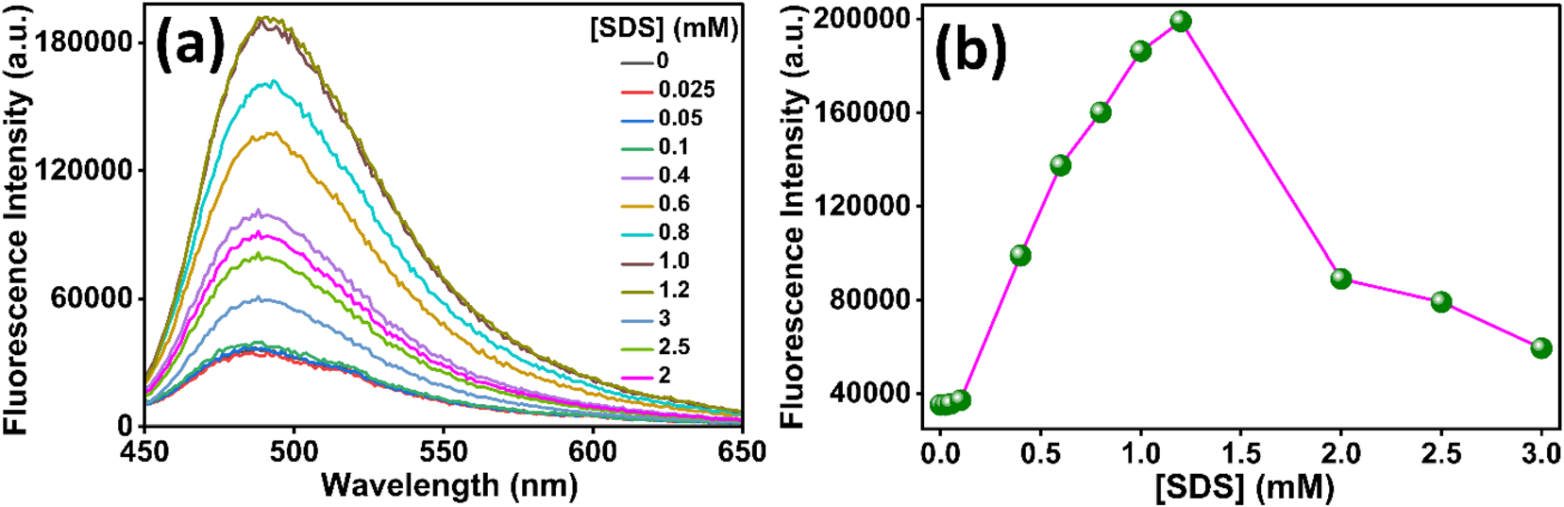
(a) Fluorescence spectra of ThT in EMb bioconjugate treated with SDS of different concentrations (*λ*_ex_ = 440 nm). (b) Fluorescence intensity of ThT in EMb bioconjugate treated with SDS of different concentrations at 485 nm peak maximum. [EMb bioconjugate] = 10.00 µM, [AuNPs] = 0.0124 µM, [ThT] = 10.00 µM, and [SDS] = 0.00 to 3.00 mM in the acetate buffer medium of pH = 4.5.

Detailed measurement methods are described in Section S1.2.8. ThT is a well-established fluorescent probe used to identify amyloid fibrils. Upon binding to amyloids, ThT exhibits enhanced emission at approximately 485 nm upon excitation at 440 nm, making it useful for confirming fibril formation.^5,34^ In this work, the assay was used to determine whether SDS-induced EMb bioconjugate aggregates possess ordered amyloid fibrils or are non-fibrillar in nature (Fig. 5a). ThT fluorescence is increased from 0.4 to 1.2 mM of SDS, with a maximum at 1.2 mM (Fig. 5b), confirming the presence of ordered cross β-rich secondary structure in the aggregates at this concentration, although shifted to a broader range than in native EMb by showing the peak maxima at ∼490 nm in all the states. This shift reflects heterogeneous nucleation and growth of fibrillar structures on the AuNPs surfaces. Notably, at 0.8 mM SDS, the turbidity is found to be maximum. However, the ThT fluorescence is not maximum at this concentration. These results clarify the difference between the types of aggregates at two different ranges of SDS concentrations. A decrease in ThT fluorescence at 2.0 mM SDS corresponds to the onset of micelle-mediated defibrillation due to the solubilization of EMb bioconjugate. Together, these observations demonstrate that AuNP conjugation modulates the SDS-induced aggregation pathway of EMb, broadening the fibrillation window and altering spectral signatures without eliminating the underlying aggregation and solubilization transitions.

#### 3.3.4 Far-UV CD spectra

**Fig. 6:**
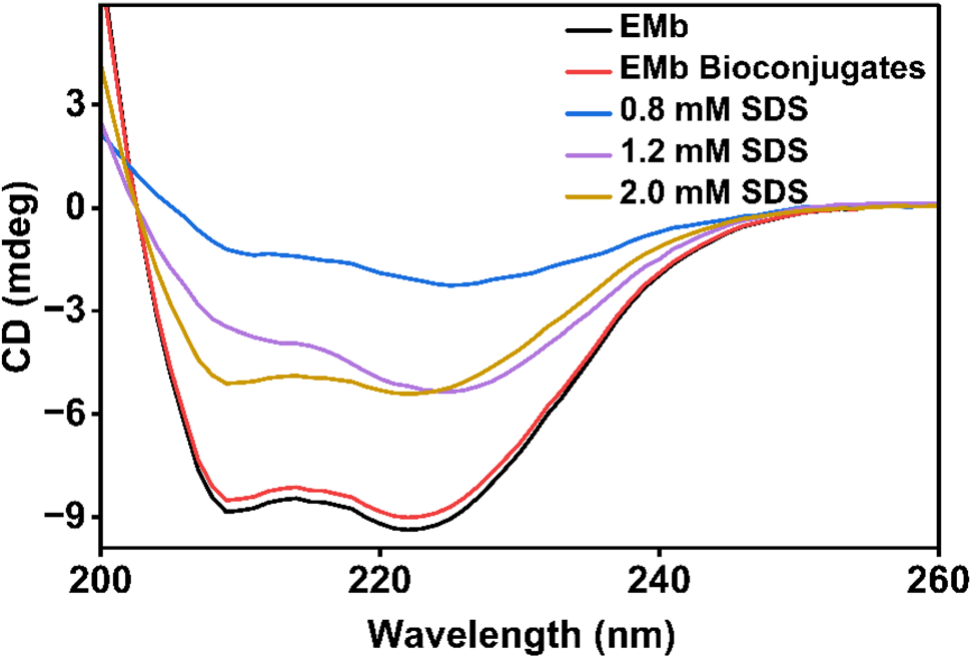
Far-UV CD spectra of EMb bioconjugates in the presence of different concentrations of SDS in acetate buffer medium at pH 4.5. [EMb bioconjugate] = 10.00 µM, [AuNPs] = 0.0124 mM.

Instrumental details are available in Section S.1.2.9. Far-UV CD measurements are used to monitor changes in the secondary structure of the EMb-AuNP bioconjugates by incubating them with SDS at various concentrations at pH 4.5. Native EMb and untreated bioconjugates both exhibited characteristic α-helical minima at 208 nm and 222 nm (Fig. 6), demonstrating that AuNP conjugation doesn't affect the protein's native secondary structure much. Increasing SDS concentrations revealed concentration-dependent conformational transitions. At 0.8 mM SDS, a complete loss of α-helical signal was observed, which correlates with amorphous aggregation (ThT-negative). At 1.2 mM SDS, the emergence of positive ellipticity at ∼225 nm indicates cross-β-sheet formation, consistent with amyloid-like fibril development. At 2.0 mM SDS, restoration of α-helical content was observed, indicating the partial formation of a native-like structure. The % changes in secondary structure calculated from the CD spectral data given in Table S1 match these observations.

#### 3.3.5 HR-TEM imaging of aggregates

**Fig. 7:**
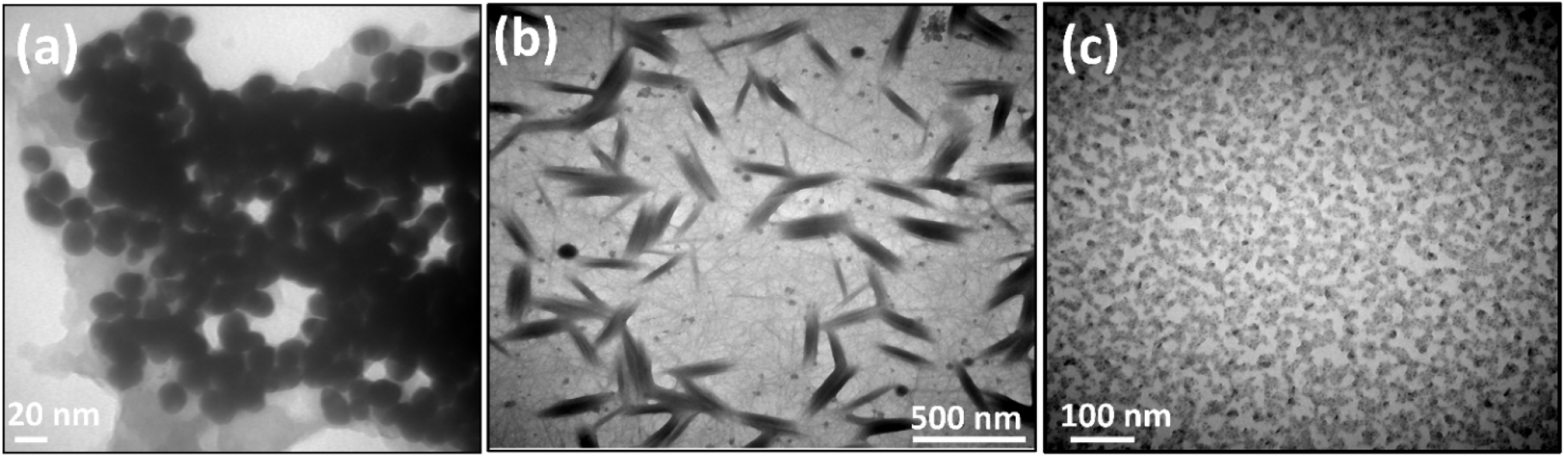
HR-TEM images of EMb-bioconjugates treated with (a) 0.8 mM SDS, (b) 1.2 mM SDS, and (c) 2.0 mM SDS. [EMb bioconjugate] = 10.00 µM, [AuNPs] = 0.0124 µM.

HR-TEM images were recorded to visually track SDS-induced morphological transitions in the EMb-AuNP bioconjugates (Fig. 7). Without SDS, the system showed uniformly distributed bioconjugates with no visible protein clustering, confirming good dispersibility of the bioconjugates (Fig. 2a). At 0.8 mM SDS, distinct bioconjugated protein aggregates were detected (Fig. 7a), indicating surfactant-induced self-assembly stress at this concentration. At 1.2 mM SDS, the morphology shifted to elongated, fibril-like networks (Fig. 7b), corroborating the ThT assay, which showed amyloid fibril formation under these conditions. When the SDS concentration was increased to 2.0 mM, fibrillar assemblies were no longer present, and the bioconjugates appeared dispersed again (Fig. 7c), supporting the defibrillation of the bioconjugate leading to solubilization and the restoration of a native-like secondary structure.

### 3.4 Effect of AuNPs on SDS-induced structural states of bioconjugates

To understand how AuNPs influence the conformational and photophysical behaviour of bioconjugates in their SDS-induced states, we examined four well-defined EMb structural regimes: native bioconjugates (0.0 mM SDS), amorphous aggregates (∼0.8 mM SDS), amyloid-like fibrils (∼1.2 mM SDS), and native like partially refolded structure (∼2.0 mM SDS). At each state, AuNPs were incrementally introduced (0.00–0.03 µM), and we monitored UV-vis absorption, steady-state fluorescence, steady-state fluorescence anisotropy, fluorescence lifetime, and time-resolved fluorescence anisotropy to quantify the interaction strength and energy transfer efficiency (*E*_T_).

Parallel experiments with Coumarin 153 (C153) and Rhodamine 6G (Rh6G) were conducted to investigate dye-AuNP interactions within the same microenvironment.

#### 3.4.1 Steady-state intrinsic fluorescence

##### Native EMb bioconjugate (0.0 mM SDS)

**Fig. 8:**
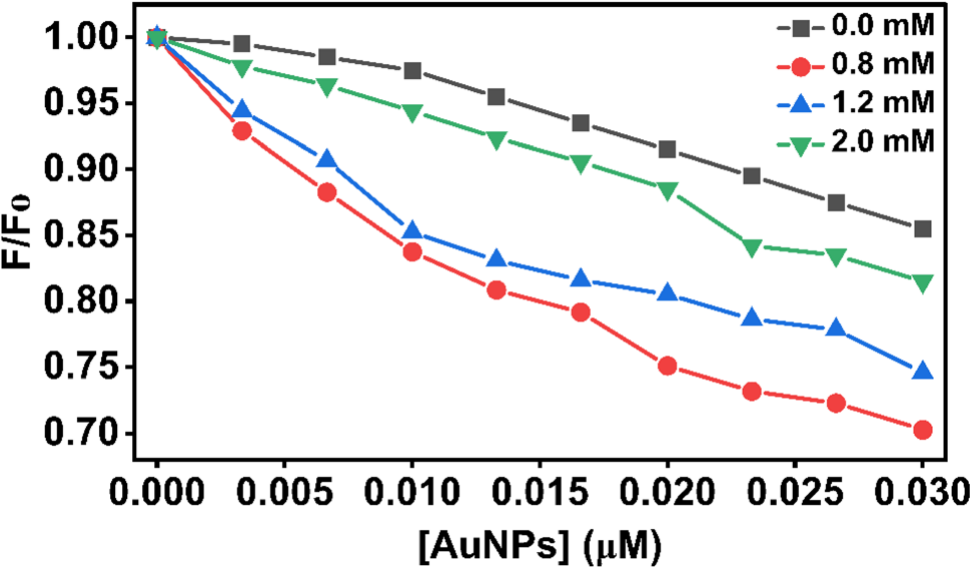
*F*/*F*_o_ plots for native EMb bioconjugate, EMb bioconjugate treated by 0.8 mM, 1.2 mM, and 2.0 mM of SDS in acetate buffer medium at pH 4.5. *λ*_ex_ = 280 nm. The range of [AuNPs] is from 0.00 to 0.03 µM, and [EMb bioconjugate] = 10.00 µM. (In *F*/*F*_o_, *F* and *F*_o_ are fluorescence intensities in the presence and absence of AuNPs, respectively).

Native EMb bioconjugate exhibited strong intrinsic fluorescence with *λ*_max_ = 335 nm at *λ*_ex_ = 280 nm, where fluorescence originated from aromatic amino acid residues embedded within the folded hydrophobic pocket of the protein. Upon gradual addition of AuNPs, a concentration-dependent decrease in fluorescence intensity was observed without any significant spectral shift (Fig. S5a). Fig. 8 illustrates changes in fluorescence intensity ratio, *F*/*F*_o_ (where *F* and *F*_o_ represent the fluorescence intensities in the presence and absence of AuNPs, respectively). From this plot, quenching with increasing AuNP concentration is clearly visible. The absence of wavelength shift suggests preservation of the native tertiary structure, while the intensity loss arises from distance-dependent non-radiative energy transfer from aromatic amino acid residues to the AuNP surface. However, compared to the aggregated states, the extent of quenching remains moderate, likely due to the burial of aromatic amino acid residues within the compact native fold, which limits close proximity between AuNPs and all these intrinsic fluorophores. Similar partial quenching behaviour has been reported for native globular proteins interacting with AuNPs, where efficient energy transfer requires solvent-exposed chromophores.^22,48^

##### Amorphous aggregates (0.8 mM SDS)

At 0.8 mM SDS, the EMb bioconjugate undergoes partial unfolding, leading to the formation of disordered amorphous aggregates. In this state, increasing the AuNP concentration resulted in markedly greater fluorescence quenching than with the native protein, as shown in Fig. S5b and 8. Partial unfolding exposes hydrophobic domains and aromatic amino acid residues, facilitating stronger, closer interactions between these intrinsic fluorophores and AuNPs (discussed later). The disordered, amorphous nature of aggregates enables AuNPs to access multiple binding sites, thereby facilitating efficient non-radiative energy transfer. Consequently, this state exhibits the maximum fluorescence quenching (Fig. 8) among all SDS-induced structural states, highlighting the critical role of protein disorder and accessibility in AuNPs-mediated quenching.

##### Amyloid fibrils (1.2 mM SDS)

At 1.2 mM SDS, EMb bioconjugate adopts an ordered amyloid fibril enriched in cross-β secondary structure. Although fibril formation perturbs the native fold, the highly ordered architecture sequesters a significant fraction of aromatic amino acid residues within the fibril core. Upon increasing AuNP concentrations, fluorescence quenching is observed, but it is less pronounced than in the amorphous aggregate state (Fig. S5c and 8). AuNPs predominantly associate with the fibril surface, resulting in a larger average donor–acceptor separation and reduced energy-transfer efficiency (discussed later). This behaviour is consistent with previous reports demonstrating partial protection of fluorophores within amyloid cores, leading to moderate AuNP-induced quenching rather than complete fluorescence loss.^49^

##### Partially refolded state (2.0 mM SDS)

At 2.0 mM SDS, above the critical micelle concentration, EMb bioconjugate undergoes solubilization and adopts a partially refolded native like α-helical conformation. In this state, fluorescence intensity shows partial recovery relative to the aggregated states (Fig. S5d and 8), although AuNP-induced quenching persists. The extent of quenching is lower than that observed for amorphous aggregates and amyloid fibrils (Fig. S5d and 8). As SDS molecules form micelles in the solution, the protein-solvent contacts are regained, helping the protein to refold back. Protein's chromophore burial increases as the hydrophobic regions get shielded, thereby limiting close AuNPs-aromatic amino acids interactions and reducing non-radiative energy transfer efficiency (discussed later). This observation further supports the conclusion that protein compactness and microenvironmental shielding are key factors in governing AuNPs-mediated fluorescence quenching.

#### 3.4.2 Intrinsic fluorescence lifetime measurements

The fluorescence intensity decay measurement methods are described in Section S1.2.2.^16,50^ Fluorescence intensity decay profiles of EMb-AuNP bioconjugates at different concentrations of AuNPs in the absence and presence of SDS were recorded, and all of them have shown tri-exponential decays, indicating the presence of multiple emissive populations of the aromatic amino acid residues arising from heterogeneous microenvironments and variable proximity to AuNP surfaces, as given in Table S2. Native EMb bioconjugate (0.0 mM SDS) exhibited tri-exponential components dominated by a longer average lifetime (∼2.7 ns) contribution (Table S2), consistent with a compact folded structure in which aromatic amino acid residues remain largely buried within the hydrophobic pocket of the protein. Upon increasing the AuNPs concentration, the shortest-lifetime component showed a relative increase in amplitude, reflecting the emergence of AuNP-coupled aromatic amino acid populations. However, the average lifetime reduction remained moderate, indicating restricted fluorophore accessibility in the native fold.

In the amorphous aggregate state (0.8 mM SDS), a pronounced redistribution of lifetime amplitudes was observed (Table S2). The shortest-lifetime component became dominant with increasing AuNP concentration, accompanied by a substantial reduction in the amplitudes of the longer-lifetime components. This behaviour arises from partial unfolding and structural disorder in amorphous aggregates, which expose aromatic amino acid residues and allow close approach to AuNP surfaces, resulting in highly efficient non-radiative energy transfer (discussed later). The tri-exponential decay reflects strong microenvironmental heterogeneity within the disordered aggregates, and this state exhibits the shortest average lifetime among all SDS-induced conformations.

Amyloid fibrils formed at 1.2 mM SDS also displayed tri-exponential decay behavior; however, the relative contribution of longer lifetime components remained significant even at higher AuNP concentrations (Table S2). The ordered cross-β architecture of amyloid fibrils sequesters a fraction of aromatic amino acid residues within the fibril core, limiting direct AuNPs interaction. Consequently, although fluorescence quenching and lifetime shortening occur, the average lifetime remains longer than that observed for amorphous aggregates.

In the partially refolded, native-like state (2.0 mM SDS), partial recovery of the longer-lived components was observed (Table S2). SDS micelle formation stabilizes α-helical structures and shields hydrophobic regions, increasing the average distance (discussed later) between aromatic amino acid residues and AuNPs. As a result, non-radiative decay pathways are suppressed relative to aggregated states, and the average fluorescence lifetime approaches that of the native EMb bioconjugate.

#### 3.4.3 Steady-state fluorescence anisotropy of intrinsic fluorophore

The method of steady-state fluorescence anisotropy measurement is available in Section S1.2.2. Steady-state fluorescence anisotropy measurements were employed to probe overall rotational mobility and microenvironmental rigidity of aromatic amino acid residues in different SDS-induced conformational states. Steady-state fluorescence anisotropy values for intrinsic fluorophores in different states are given in Table S3. Native EMb bioconjugate displayed relatively high fluorescence anisotropy values, consistent with restricted rotational freedom within a compact globular structure, and fluorescence anisotropy decreased slightly with increasing AuNP concentration.

Almost the same fluorescence anisotropy was observed for the amorphous aggregate state (0.8 mM SDS) as noted in the native EMb bioconjugate, except for the higher AuNP concentrations (Table S3). No significant change in the flexibility of the protein segment containing amino acid residues has been noted. However, in this state of the bioconjugate, efficient coupling between aromatic amino acid residues and AuNPs, leading to greater energy transfer as explained above, could be due to its open structure.

Amyloid fibrils (1.2 mM SDS) also exhibited almost the same fluorescence anisotropies of the protein segment containing aromatic amino acid residues as amorphous aggregates (Table S3). The segmental motions in the protein interior are not changed significantly compared to the amorphous state. In the partially refolded, native-like state (2.0 mM SDS), fluorescence anisotropy values increased slightly, consistent with structural reorganization into more compact α-helical conformations and reduced conformational freedom (Table S3). In all systems, fluorescence anisotropy decreases with increasing AuNP concentration. Results indicate that AuNPs introduce some degree of flexibility into the system.

#### 3.4.4 Radiative and non-radiative rate constants and energy transfer analysis for intrinsic fluorophores

To further elucidate the mechanism of AuNP-induced fluorescence quenching across the SDS-induced structural states of EMb-AuNP bioconjugates, radiative (*k*_r_) and non-radiative (*k*_nr_) decay rate constants were estimated using eqn (S7) and (S8),^51^ respectively, and given in Table S2.

**Fig. 9:**
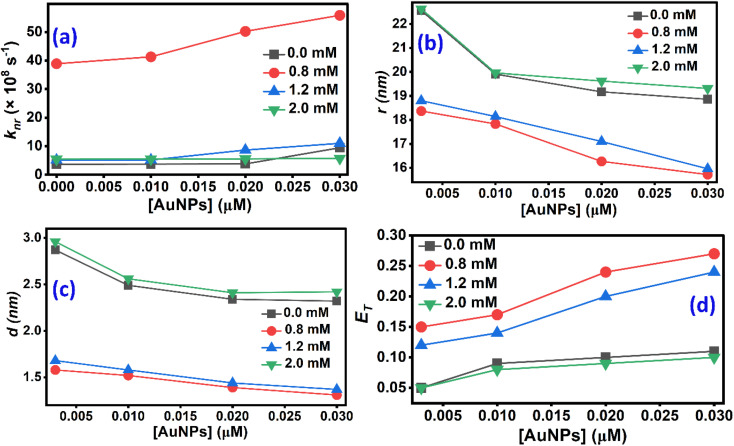
(a) *k*_nr_, (b) *r* (FRET), (c) *d* (NSET), and (d) *E*_T_ for native EMb bioconjugate (0.0 mM SDS), EMb bioconjugate treated with 0.8 mM, 1.2 mM, and 2.0 mM of SDS with increasing AuNPs concentration in acetate buffer medium of pH 4.5. [EMb bioconjugate] = 10.00 µM, [AuNPs] = 0.003 to 0.030 µM. Standard deviations are given in Table S4.

For native EMb bioconjugates (0.0 mM SDS), the radiative (*k*_r_) and non-radiative (*k*_nr_) decay rate constants remain nearly invariant upon increasing AuNP concentration at low concentration range, indicating that the intrinsic emissive properties of aromatic amino acid residues are preserved (Table S2 and Fig. 9a). In contrast, a moderate increase in *k*_nr_ was observed at a higher concentration of AuNPs, consistent with the introduction of an additional non-radiative decay pathway arising from distance-dependent energy transfer to the AuNP surface. The limited rise in *k*_nr_ reflects restricted accessibility of intrinsic fluorophores, resulting in moderate energy-transfer efficiency.

In the amorphous aggregate state (0.8 mM SDS), a substantial increase in *k*_nr_ was observed. The *k*_nr_ was also increased significantly with increasing AuNPs concentration, while *k*_r_ remained largely unchanged (Table S2 and Fig. 9a). This pronounced enhancement of the non-radiative decay rate arises from partial unfolding and structural disorder of amorphous aggregates, which expose aromatic amino acid residues and facilitate close approach to AuNPs. The dominance of *k*_nr_ in this state confirms highly efficient energy transfer, consistent with NSET (discussed later), which is strongly distance-dependent and favoured in disordered protein assemblies.

Amyloid fibrils formed at 1.2 mM SDS exhibited comparatively lower *k*_nr_ than that in the amorphous aggregate state (Table S2 and Fig. 9a). An increase in *k*_nr_ upon AuNPs addition was also noted; however, the magnitude of this increase was smaller than that observed for amorphous aggregates (Table S2 and Fig. 9a). The ordered cross-β-sheet architecture shields a fraction of aromatic amino acid residues within the fibril core, increasing the average donor–acceptor distance and limiting energy-transfer efficiency (discussed later). Consequently, *k*_nr_ values remain intermediate between those of amorphous aggregates and native EMb bioconjugate.

In the partially refolded native-like state (2.0 mM SDS), *k*_nr_ decreased relative to aggregated and amyloid fibril states (Table S2 and Fig. 9a). SDS micelle formation stabilises α-helical conformations and shields hydrophobic regions, thereby suppressing AuNP-induced non-radiative decay.

Overall, the observed trends in *k*_r_ and *k*_nr_ clearly demonstrate that AuNP-induced quenching across SDS-induced amorphous aggregates > amyloid fibrils > refolded native-like state ≈ native EMb bioconjugate, consistent with steady-state fluorescence and fluorescence lifetime analyses. Alterations in the conformational states of EMb predominantly affect non-radiative energy transfer efficiency rather than intrinsic radiative emission. The hierarchy of energy-transfer efficiency data is provided in Table S4. The detailed method for calculating energy transfer parameters based on FRET and NSET mechanisms is provided in Section S1.3 of the SI. The data in Table S4 and Fig. 9 show that, in general, with increasing AuNP concentration, the donor–acceptor distance *r* (FRET) (Fig. 9b) and *d* (NSET) (Fig. 9c) decrease, and the energy transfer efficiency (*E*_T_) (Fig. 9d) increases as expected. Notably, *r* and *d* are both shorter, and *E*_T_ values are larger in amorphous aggregates and fibrillar states compared to native and partially refolded states (Table S4 and Fig. 9b–d). As expected, *r*, *d*, and *E*_T_ values are nearly identical in the native and partially refolded states of the bioconjugates (Table S4 and Fig. 9b–d). *E*_T_ values are consistent with the donor–acceptor distances. These results are consistent with the radiative and non-radiative rate constants discussed above, as they reflect the rate of energy-transfer processes controlled by the donor–acceptor distance.

### 3.5 Probing SDS-induced structural states of EMb-AuNP bioconjugates using extrinsic fluorescence of dyes

To understand microenvironmental changes and energy-transfer processes during SDS-induced structural transitions of EMb-AuNP bioconjugates, two spectrally distinct fluorescent probes were employed: Coumarin-153 (C153), a polarity-sensitive dye preferentially residing in hydrophobic or interior regions, and Rhodamine-6G (Rh6G), a cationic dye as a surface-associated, interfacial probe. The distinct photophysical responses of these dyes enable differentiation between interior-confined and surface-exposed regions across native, amorphous aggregate, amyloid fibril, and refolded states.

#### 3.5.1 Steady-state fluorescence of dyes

**Fig. 10:**
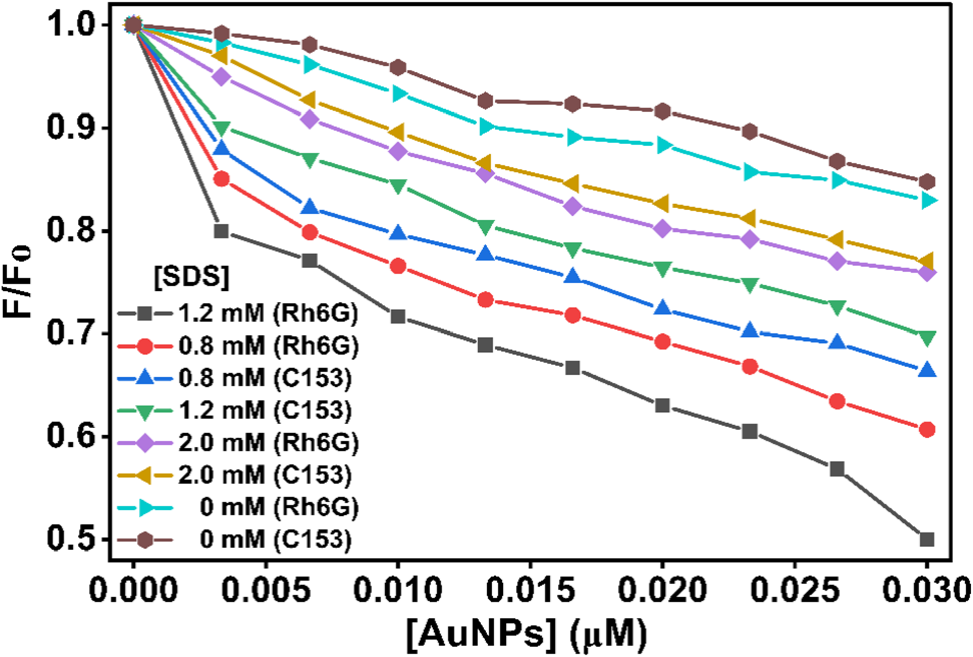
*F*/*F*_o_ plots of C153 and Rh6G in native EMb bioconjugate (0.0 mM SDS), EMb bioconjugate treated by 0.8 mM of SDS, 1.2 mM of SDS, and 2.0 mM of SDS in the acetate buffer of pH 4.5. *λ*_ex_ = 409 nm (C153), *λ*_ex_ = 510 nm (Rh6G). The range of [AuNPs] is from 0.00 to 0.03 µM, and [EMb bioconjugate] = 10.00 µM. [C153] and [Rh6G] = 0.5 µM. (In *F*/*F*_o_, *F* and *F*_o_ are fluorescence intensities in the presence and absence of AuNPs).

Steady state fluorescence spectra of dyes C153 and Rh6G in EMb-AuNP bioconjugate systems at 0.0 mM, 0.8 mM, 1.2 mM, and 2.0 mM of SDS have been recorded. The spectra for C153 and Rh6G are given in Fig. S6 and S7, respectively. Fig. 10 shows changes in the fluorescence intensity ratio, *F*/*F*_o_ (where *F* and *F*_o_ are the fluorescence intensities in the presence and absence of AuNPs, respectively), for both dyes as the AuNPs concentration increases across various systems.

In the native EMb-AuNP bioconjugate state, C153 exhibits relatively high fluorescence intensity (Fig. S6a and 10), indicating preferential localization within hydrophobic or partially buried regions of the protein matrix. In contrast, Rh6G displays moderate quenching (Fig. S7a and 10), due to its proximity to the protein-AuNP interface, where surface-bound AuNPs facilitate non-radiative energy dissipation *via* NSET.

Upon SDS addition leading to amorphous aggregate formation, fluorescence quenching is significantly enhanced for both dyes, with a more pronounced decrease observed for Rh6G compared to C153 (Fig. S6b, S7b, and 10). Partial protein unfolding at this stage exposes hydrophobic residues and creates heterogeneous, solvent-accessible environments, increasing dye-AuNP proximity. While C153 is displaced from its native buried sites into more polar regions, Rh6G remains strongly coupled to exposed aggregate surfaces, leading to greater fluorescence quenching.

In the amyloid fibril state, C153 fluorescence shows relative stabilization or partial recovery compared to amorphous aggregates (Fig. S6c and 10). This behavior is attributed to sequestration of C153 within cross-β-sheet-rich fibrillar interiors that restrict solvent and AuNPs access and reduce the non-radiative decay rate. Conversely, Rh6G undergoes the strongest quenching (Fig. S7c and 10) in this state, consistent with its preferential adsorption along extended fibril surfaces, where AuNPs remain closely associated.

At higher SDS concentrations corresponding to a partially refolded or native-like state, fluorescence intensities of both dyes partially recover, reflecting reorganization of protein structure and reduced accessibility of AuNPs to dye molecules (Fig. S6d, S7d, and 10).

#### 3.5.2 Fluorescence lifetimes of dyes, and FRET/NSET parameters

The fluorescence intensity decay parameters of C153 (Table 1) across all structural states exhibit triexponential decay, reflecting the presence of multiple dye populations residing in distinct microenvironments, with differences in polarity, rigidity, and proximity to AuNPs. Whereas Rh6G (Table 2) has followed a biexponential model across all states, consistent with its localization in two dominant environments: surface-bound dye molecules proximal to AuNPs and a population weakly associated with the protein matrix. To compare the energy transfer efficiencies between a dye and AuNPs through the NSET/FRET mechanism in different states of bioconjugates in the presence of varying concentrations of AuNPs, the radiative (*k*_r_) and non-radiative (*k*_nr_) rate constants of C153 and Rh6G have been calculated using eqn (S7) and (S8), respectively, and are tabulated in Tables 1 and 2 for these two dyes, respectively.

**Table 1:** Excited singlet state lifetimes (*τ*_1_, *τ*_2_, *τ*_3_) and corresponding weights (*α*_1_, *α*_2_, *α*_3_), average lifetime (〈*τ*_f_〉), fluorescence quantum yield (*ϕ*_f_), radiative (*k*_r_) and non-radiative (*k*_nr_) rate constants of C153 in native EMb bioconjugate, EMb bioconjugate treated with 0.8 mM, 1.2 mM, and 2.0 mM of SDS in acetate buffer medium at pH 4.5. [EMb bioconjugate] = 10.00 µM, [C153] = 0.5 µM, [AuNPs] = 0.00 to 0.03 µM. *λ*_ex_ = 405 nm, *λ*_em_ = 530 nm

C153											
[AuNP] (µM)	*α*_1_	*τ*_1_ (ps)	*α*_2_	*τ*_2_ (ps)	*α*_3_	*τ*_3_ (ps)	〈*τ*_f_〉 (ps)	*χ* ^2^	*ϕ*_f_	*k*_r_ (×10^6^ s^−1^)	*k*_nr_ (×10^8^ s^−1^)
**0.0 mM SDS**											
0.00	0.10 ± 0.01	534 ± 59	0.83 ± 0.03	173 ± 132	0.07 ± 0.01	4455 ± 137	1796 ± 125	1.06	0.695	386.90	1.70
0.01	0.11 ± 0.03	636 ± 79	0.87 ± 0.03	1757 ± 154	0.02 ± 0.01	6236 ± 189	1723 ± 146	1.12	0.690	400.40	1.79
0.02	0.07 ± 0.01	392 ± 92	0.88 ± 0.03	1670 ± 127	0.05 ± 0.03	4338 ± 153	1714 ± 125	1.00	0.580	338.40	2.45
0.03	0.07 ± 0.01	459 ± 83	0.89 ± 0.03	1657 ± 118	0.04 ± 0.01	4242 ± 145	1676 ± 116	1.15	0.510	304.30	2.92

**0.8 mM SDS**											
0.00	0.76 ± 0.04	207 ± 88	0.18 ± 0.01	1135 ± 107	0.06 ± 0.01	2721 ± 109	536 ± 92	1.16	0.0015	2.80	18.63
0.01	0.80 ± 0.03	212 ± 69	0.16 ± 0.01	1319 ± 111	0.04 ± 0.02	2906 ± 112	509 ± 77	1.05	0.0008	1.57	19.63
0.02	0.74 ± 0.02	113 ± 29	0.20 ± 0.03	996 ± 98	0.06 ± 0.01	2740 ± 128	454 ± 48	0.98	0.0006	1.32	22.01
0.03	0.84 ± 0.01	198 ± 19	0.13 ± 0.02	1156 ± 56	0.03 ± 0.01	2788 ± 123	399 ± 26	1.15	0.0001	0.25	25.06

**1.2 mM SDS**											
0.00	0.17 ± 0.02	758 ± 88	0.37 ± 0.02	2745 ± 89	0.46 ± 0.02	4901 ± 125	3389 ± 105	1.01	0.0215	6.34	2.89
0.01	0.21 ± 0.02	833 ± 78	0.34 ± 0.01	2811 ± 79	0.45 ± 0.01	4917 ± 109	3341 ± 92	1.02	0.0188	5.62	2.94
0.02	0.22 ± 0.01	845 ± 94	0.38 ± 0.02	2833 ± 109	0.40 ± 0.02	4937 ± 119	3227 ± 109	1.00	0.0173	5.36	3.05
0.03	0.23 ± 0.03	801 ± 78	0.35 ± 0.02	2743 ± 117	0.42 ± 0.03	4876 ± 126	3181 ± 111	0.99	0.0159	4.99	3.09

**2.0 mM SDS**											
0.00	0.13 ± 0.02	548 ± 87	0.80 ± 0.02	1672 ± 107	0.07 ± 0.03	4318 ± 209	1684 ± 111	1.01	0.712	421.60	1.71
0.01	0.11 ± 0.02	462 ± 98	0.83 ± 0.03	1701 ± 118	0.06 ± 0.01	4257 ± 302	1659 ± 126	1.09	0.700	421.90	1.81
0.02	0.15 ± 0.02	598 ± 74	0.78 ± 0.04	1633 ± 127	0.07 ± 0.02	4104 ± 108	1612 ± 117	1.12	0.690	428.00	1.92
0.03	0.12 ± 0.02	489 ± 123	0.82 ± 0.07	1665 ± 153	0.06 ± 0.01	4221 ± 209	1578 ± 152	1.05	0.670	424.60	2.09

**Table 2:** Excited singlet state lifetimes (*τ*_1_, *τ*_2_) and corresponding weights (*α*_1_, *α*_2_), average lifetime (〈*τ*_f_〉), fluorescence quantum yield (*ϕ*_f_), radiative (*k*_r_) and non-radiative (*k*_nr_) rate constants of Rh6G in native EMb bioconjugate, EMb bioconjugate treated with 0.8 mM of SDS, 1.2 mM of SDS, and 2.0 mM of SDS in acetate buffer medium at pH 4.5. [EMb bioconjugate] = 10.00 µM, [Rh6G] = 0.5 µM, [AuNPs] = 0.00 to 0.03 µM. *λ*_ex_ = 510 nm, *λ*_em_ = 560 nm

Rh6G									
[AuNP] (µM)	*α*_1_	*τ*_1_ (ps)	*α*_2_	*τ*_2_ (ps)	〈*τ*_f_〉 (ps)	*χ* ^2^	*ϕ*_f_	*k*_r_ (×10^6^ s^−1^)	*k*_nr_ (×10^8^ s^−1^)
**0.0 mM SDS**									
0.00	0.03 ± 0.01	2906 ± 101	0.97 ± 0.02	3953 ± 168	3921 ± 165	1.01	0.0120	3.06	2.52
0.01	0.05 ± 0.02	2762 ± 99	0.95 ± 0.02	3965 ± 121	3905 ± 119	0.99	0.0113	2.89	2.53
0.02	0.12 ± 0.02	2846 ± 131	0.88 ± 0.01	4014 ± 153	3879 ± 150	1.01	0.0107	0.21	2.58
0.03	0.09 ± 0.01	1923 ± 137	0.91 ± 0.01	4014 ± 89	3821 ± 93	1.05	0.0100	0.18	2.61

**0.8 mM SDS**									
0.00	0.41 ± 0.03	721 ± 109	0.59 ± 0.02	3908 ± 174	2615 ± 147	1.07	0.0065	0.25	3.80
0.01	0.42 ± 0.02	764 ± 98	0.58 ± 0.03	3923 ± 150	2589 ± 128	0.99	0.0060	0.23	3.84
0.02	0.49 ± 0.02	694 ± 62	0.51 ± 0.01	3936 ± 89	2357 ± 75	0.99	0.0055	0.24	4.22
0.03	0.53 ± 0.01	640 ± 44	0.47 ± 0.01	3877 ± 56	2175 ± 49	1.02	0.0050	0.24	4.57

**1.2 mM SDS**									
0.00	0.83 ± 0.02	248 ± 32	0.17 ± 0.02	3899 ± 101	867 ± 43	1.18	0.0002	0.23	11.53
0.01	0.85 ± 0.01	269 ± 29	0.15 ± 0.03	3871 ± 89	796 ± 38	0.99	0.0002	0.25	12.56
0.02	0.85 ± 0.01	253 ± 26	0.15 ± 0.02	3889 ± 78	789 ± 33	1.17	0.0001	0.13	12.67
0.03	0.85 ± 0.03	212 ± 24	0.15 ± 0.01	3837 ± 62	762 ± 29	1.07	0.0001	0.13	13.12

**2.0 mM SDS**									
0.00	0.18 ± 0.01	2035 ± 99	0.82 ± 0.02	4226 ± 87	3822 ± 89	1.19	0.0109	2.85	2.58
0.01	0.15 ± 0.01	1534 ± 87	0.85 ± 0.02	4185 ± 109	3782 ± 105	1.12	0.0101	2.67	2.62
0.02	0.17 ± 0.02	1649 ± 76	0.83 ± 0.01	4207 ± 111	3781 ± 105	1.06	0.0095	2.51	2.63
0.03	0.17 ± 0.03	1583 ± 62	0.83 ± 0.01	4206 ± 98	3767 ± 91	1.07	0.0088	2.34	2.65

Both FRET and NSET mechanisms are applied for energy transfer between a dye (C153 or Rh6G) and AuNPs. The FRET and NSET parameters have been calculated to support the dye locations, and, based on those, the different states of EMb bioconjugates in the absence and presence of SDS have been explained. The parameters *J*, *R*_o_ (FRET), *d*_o_ (NSET), *r* (FRET), *d* (NSET), and *E*_T_ have been calculated using equations S9–S15. These values for C153 and Rh6G are given in Tables S5 and S6, respectively.

**Fig. 11:**
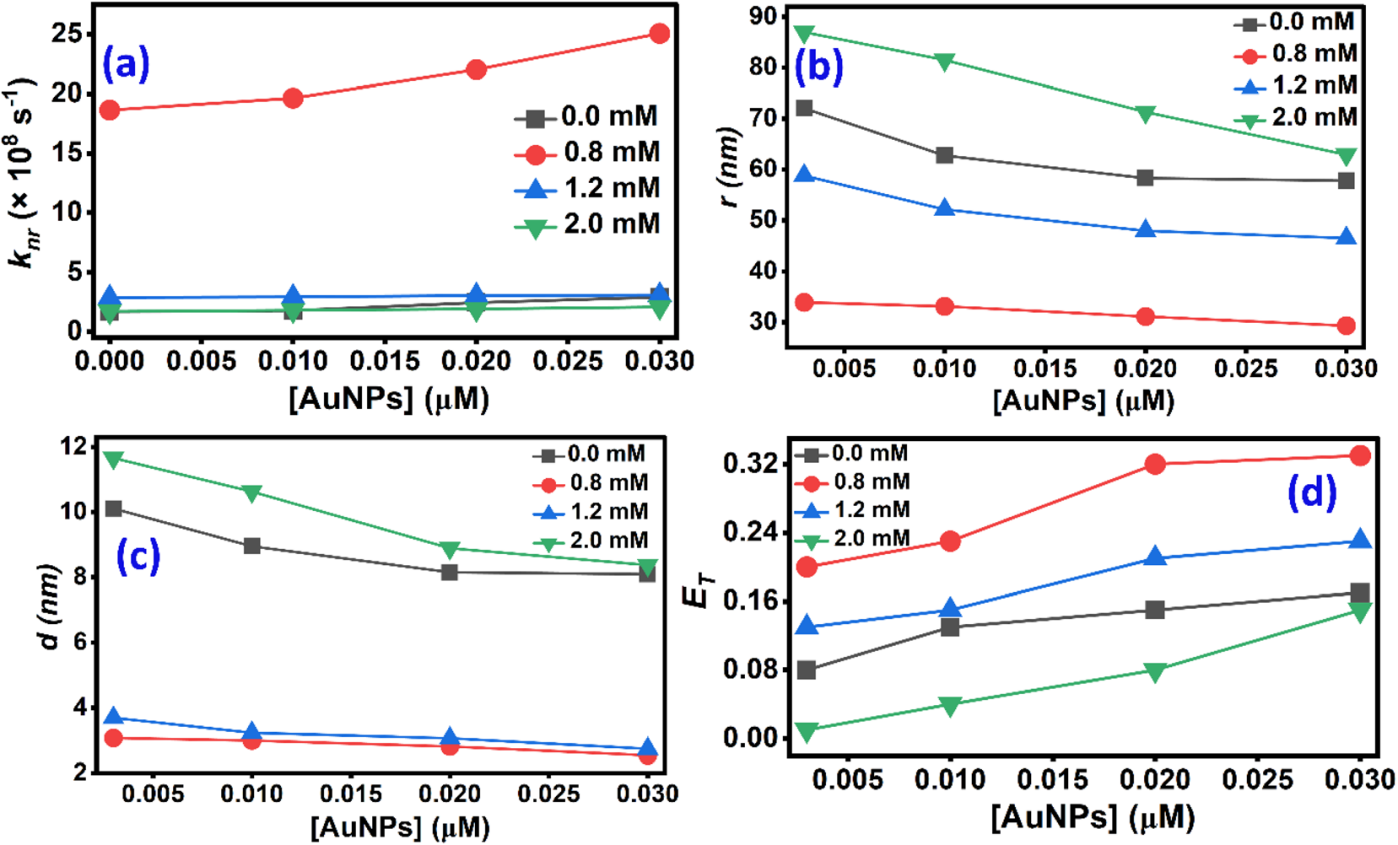
(a) *k*_nr_, (b) *r* (FRET), (c) *d* (NSET), and (d) *E*_T_ for C153 in native EMb bioconjugate (0.0 mM SDS), EMb bioconjugate treated with 0.8 mM, 1.2 mM, and 2.0 mM of SDS with increasing AuNPs concentration in acetate buffer medium at pH 4.5. [EMb bioconjugate] = 10.00 µM, [AuNPs] = 0.003 to 0.030 µM. Standard deviations are given in Table S5.

**Fig. 12:**
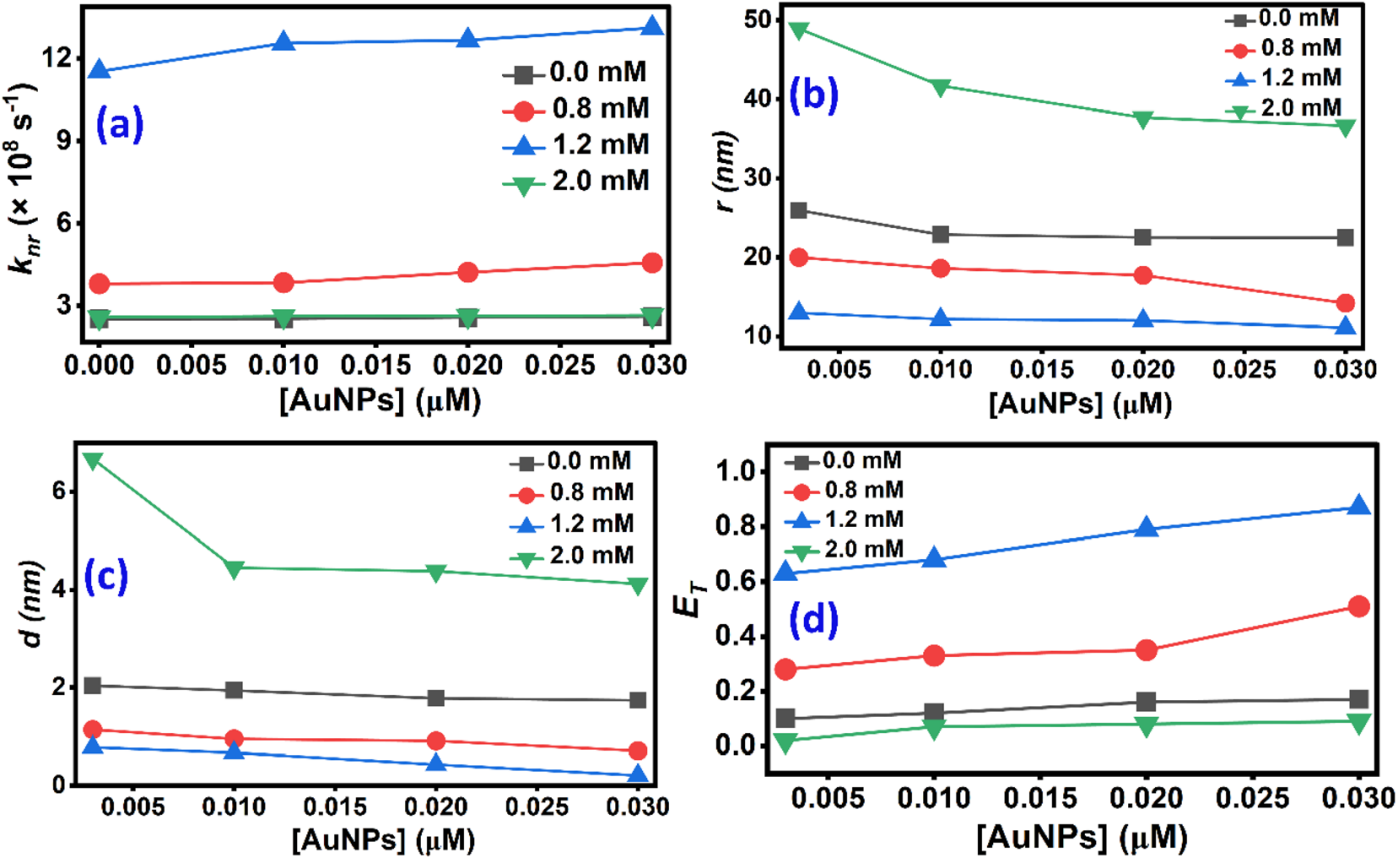
(a) *k*_nr_, (b) *r* (FRET), (c) *d* (NSET), and (d) *E*_T_ for Rh6G in native EMb bioconjugate (0.0 mM SDS), EMb bioconjugate treated with 0.8 mM, 1.2 mM, and 2.0 mM of SDS with increasing AuNPs concentration in acetate buffer medium at pH 4.5. [EMb bioconjugate] = 10.00 µM, [AuNPs] = 0.003 to 0.030 µM. Standard deviations are given in Table S6.

In the native state of bioconjugate, the component with intermediate lifetime dominates (83–89%) for C153 (Table 1). The shortest and longest components appear with amplitudes of 7–11% and 2–7%, respectively. The heterogeneity in the microenvironment is due to the different locations of C153: deep inside the protein, with the longest lifetime; towards the outer layer, with the shortest lifetime; and an intermediate-polarity region with a moderately high lifetime. However, no significant change in either the lifetimes of C153 or their amplitudes with increasing AuNP concentration rules out a very close proximity of the dye to the AuNP surfaces. The results are supported by the lowest non-radiative process rate in this case (Table 1 and Fig. 11a). The longest average C153-AuNPs distance (*r* (FRET) (Fig. 11b) and *d* (NSET) (Fig. 11c)) and lowest energy transfer efficiency (*E*_T_) (Fig. 11d) values in this state among four states excluding refolded state, as seen from Table S5 and Fig. 11, support this result. Rh6G exhibits two components with lifetimes of approximately 2.9 and 4.0 ns, with the longer one being the major component (Table 2). Although the lifetime of the longer component remains unchanged, the slight quenching of the shorter component is noted only at higher AuNP concentrations, suggesting its proximity to the AuNP surfaces. The *k*_nr_ values of Rh6G as a function of AuNPs concentrations are plotted in Fig. 12a. As expected, the *k*_nr_ values for Rh6G are mostly greater than those for C153 (Tables 1, 2 and Fig. 11a, 12a). The substantially shorter Rh6G-AuNPs distance seen in Table S6 supports this observation. The variations in *r* (FRET), *d* (NSET), and *E*_T_ with increasing AuNP concentration for Rh6G are shown in Fig. 12b–d, respectively. The decrease in donor–acceptor distance (*r* and *d*) and the concomitant increase in *E*_T_ with increasing AuNPs concentrations for both dyes support the energy-transfer mechanism (Fig. 11 and 12).

In the amorphous aggregate state, both dyes exhibit a marked increase in the contribution of short-lived components (Tables 1 and 2), indicating enhanced non-radiative decay pathways (Fig. 11a and 12a) resulting from closer dye-AuNP distances, which confirms efficient FRET/NSET from dye molecules. From the data in Table S5 and Fig. 11b–d for C153 and Table S6 and Fig. 12b–d for Rh6G, it can be seen that the dye-AuNPs distance is shorter and *E*_T_ is higher in the amorphous aggregate state than in the native bioconjugate state. The non-radiative rate constants (*k*_nr_) of C153 and Rh6G, given in Table 1 (Fig. 11a) and Table 2 (Fig. 12a), respectively, are found to be enhanced due to greater energy transfer. The lifetimes of all three components of C153 are substantially shortened as compared to native bioconjugates. The significant increase in the amplitude of the shortest component is compensated by a concomitant decrease in the amplitude of the component with intermediate lifetime. There is almost no change in the amplitude of the longest component. It implies that the component with the intermediate lifetime is mostly converted to the shortest-lived component, indicating the opening of the protein's moderately inner matrix. This result is supported by the change in Rh6G lifetimes. There is a significant enhancement in the amplitudes of the shorter component, accompanied by a substantial reduction in the lifetimes. The lifetime of the longer component remains almost the same, with a decrease in the amplitudes. The average lifetimes of both dyes decrease with increasing AuNP concentration.

In amyloid fibrils, C153 shows a redistribution toward longer lifetime components compared to amorphous aggregates (Table 1), reflecting confinement within ordered fibrillar aggregates' interiors that limit non-radiative relaxation pathways (Fig. 11a). The lifetimes of all components and average lifetimes are enhanced with substantially increased amplitude of the longest component (present deep inside the protein) and decreased amplitude of the shortest component. Table 1 and Fig. 11a show that the *k*_nr_ values of C153 in amyloid fibrils are lower than those in the amorphous aggregate state. Thus, C153 molecules present in the fibrillar interiors of cross-β secondary structures experience restriction from their exposure to the AuNPs, reducing the non-radiative decay rate constant. The longer *r* (Table S5 and Fig. 11b) and *d* (Table S5 and Fig. 11c) values, and the lower *E*_T_ (Table S5 and Fig. 11d) for C153 in this state compared to the amorphous aggregate state, strengthen the above hypothesis. Results also support the view that amyloid fibrils are ordered aggregates rather than an amorphous aggregate state. In contrast to C153, Rh6G lifetimes, however, remain dominated by short components (Table 2), consistent with persistent surface localization and efficient AuNP-mediated quenching, yielding significantly higher *k*_nr_ values (Table 2 and Fig. 12a). Results are supported by the substantial increase in *E*_T_ (Table S6 and Fig. 12d) and the decrease in *r* and *d* values (Table S6 and Fig. 12b, c). The accessibility of AuNPs by Rh6G is supported by the fact that the fast component's lifetime and average lifetime are decreased with increasing concentration of AuNPs. The results are indeed consistent with substantial fluorescence quenching (Fig. 10), indicating a significant increase in the non-radiative rate constant (Table 2 and Fig. 12a) as compared to the amorphous aggregate state.

At 2.0 mM SDS, the lifetimes are almost equal to the native-state lifetime with similar amplitudes of all components. Similar trends in the values of *k*_nr_, *r*, *d*, and *E*_T_ for both dyes with increasing AuNPs concentration (Fig. 11 and 12) have been observed, as noted for native EMb bioconjugates. However, there are significant differences in the values of these quantities between native and partially refolded EMb bioconjugates. In contrast, for an intrinsic fluorophore, these differences are much smaller. This could be due to the fact that at a 2.0 mM concentration of SDS, micelles are formed by the surfactant molecules in the solution, and there are chances that some C153 or Rh6G molecules will bind with the micelles as well. In addition, the protein is partially refolded. These factors will lead to differences in the values of the above-mentioned quantities between the native and partially refolded states. However, in the case of intrinsic fluorophores, these differences are only due to partial refolding, as fluorophores cannot bind with micelles, as they are covalently linked with the protein.

Notably, upon comparison of donor (dye/intrinsic fluorophore)–acceptor (AuNPs) distance among two dyes and intrinsic fluorophores, one can see that C153 is located farthest away from AuNPs (Tables S4–S6 and Fig. 9, 11, and 12), which justifies the use of C153 as a reporter for the interior of the protein. Results also indicate the protein's aromatic amino acid residues are not buried too deeply inside the protein. It is worth noting that the D–A distance calculated using the NSET mechanism will be more reliable than that from the FRET method, as in some cases the latter method yields a distance outside the acceptable range, *i.e.*, 90 A°.

These observations collectively indicate that structural ordering in amyloids selectively protects interior probes while amplifying surface-localized energy transfer. The differing decay models for C153 and Rh6G thus directly reflect their interior *versus* surface localization and validate their use as complementary probes.

#### 3.5.3 Steady-state fluorescence anisotropy of dyes

Steady-state fluorescence anisotropy measurements further support differences in dye confinement and mobility. The data in Table S3 show that, in the native state, C153 exhibits higher anisotropy values than Rh6G, consistent with restricted rotational freedom within the protein interior. Rh6G exhibits lower fluorescence anisotropy due to its surface-associated, dynamically accessible environment.

Amorphous aggregate formation increases fluorescence anisotropy for both dyes (Table S3), indicating restricted rotational mobility due to structural confinement and heterogeneous environments. C153 shows a particularly sharp increase in fluorescence anisotropy, reflecting a comparatively more rigid interior than exterior of the protein. These results contrast with the steady-state fluorescence anisotropy of intrinsic probes, which is slightly lower than that in the native state. It could be that protein segments attached to aromatic amino acids are more flexible than the environment around extrinsic dyes in the amorphous aggregate state.

In contrast to the amorphous aggregate state, amyloid fibril formation results in a decrease in fluorescence anisotropy for C153, consistent with a comparatively flexible interior of the ordered cross-β secondary structure (Table S3). Rh6G shows substantial anisotropy enhancement, suggesting a comparatively rigid exterior. The steady-state fluorescence anisotropies of both dyes are nearly identical for native and refolded bioconjugates (Table S3).

#### 3.5.4 Time-resolved fluorescence anisotropy

**Fig. 13:**
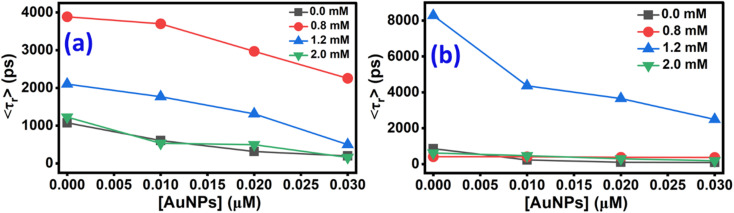
Variation of 〈*τ*_r_〉 of (a) C153 and (b) Rh6G as a function of AuNPs concentrations in native EMb bioconjugate (0.0 mM SDS), EMb bioconjugate treated with 0.8 mM of SDS, EMb bioconjugate treated with 1.2 mM of SDS, and EMb bioconjugate treated with 2.0 mM of SDS in acetate buffer medium at pH 4.5. [EMb bioconjugate] = 10.00 µM, [AuNPs] = 0 to 0.03 µM, [C153] = 0.5 µM, [Rh6G] = 0.5 µM, For C153 *λ*_ex_ = 405 nm, *λ*_em_ = 530 nm, and for Rh6G, *λ*_ex_ = 510 nm, *λ*_em_ = 560 nm. Standard deviations are given in Tables 3 and 4.

The method of fluorescence anisotropy decay measurement is available in Section 1.2.2. Time-resolved fluorescence anisotropy decay measurements reveal multicomponent rotational correlation times for both dyes. The time-resolved fluorescence anisotropy values have been calculated using eqn (S4).^52^ The rotational relaxation times have been calculated after fitting the anisotropy decay data to eqn (S5). For both dyes, bi-exponential fittings have been performed, yielding two rotational components with short (*τ*_1r_) and long (*τ*_2r_) rotational relaxation times. The average rotational relaxation times (〈*τ*_r_〉) have been calculated using eqn (S6). All anisotropy decay parameters for C153 and Rh6G in various states have been tabulated in Tables 3 and 4, respectively. The variations of 〈*τ*_r_〉 as a function of AuNPs concentrations for C153 and Rh6G have been displayed by Fig. 13a and b, respectively. For C153, the slow motion is ascribed to the component present deep inside the protein, whereas the fast motion is due to the component located in the comparatively outer region of the protein. However, for Rh6G, depolarisation is overall contributed by the dye molecules near the protein surface, giving shorter rotational relaxation times. In the native state of bioconjugates, the faster rotational motion is the major contributor to depolarisation of both dyes. For C153, the slow component is much longer than that of Rh6G (Tables 3 and 4, respectively), resulting in slower average rotational correlation times than for Rh6G, consistent with restricted interior motions. This conclusion is based on the fact that C153 is mostly located in a comparatively nonpolar region. The *r*_o_ values that symbolise the limiting anisotropy are closer to 0.40 (the maximum value of *r*_o_) for C153 than that for Rh6G. These results support the idea that Rh6G resides in a comparatively flexible environment. In other words, the protein interior is more rigid than its exterior. For both C153 and Rh6G, at all states of bioconjugates, a decrease in 〈*τ*_r_〉 with increasing AuNPs concentration has been observed (Fig. 13a and b, respectively). It depicts AuNPs-induced enhancement in the flexibility of the environment around a dye.

**Table 3:** Rotational relaxation times (*τ*_1r_, *τ*_2r_) and corresponding weights (*α*_1r_, *α*_2r_), average rotational relaxation time (〈*τ*_r_〉) and limiting anisotropy (*r*_o_) of C153 in native EMb bioconjugate (0.0 mM SDS), EMb bioconjugate treated with 0.8 mM of SDS, EMb bioconjugate treated with 1.2 mM of SDS, and EMb bioconjugate treated with 2 mM of SDS in the acetate buffer medium at pH 4.5. [EMb bioconjugate] = 10.00 µM, [C153] = 0.5 µM, [AuNPs] = 0 to 0.03 µM. *λ*_ex_ = 405 nm, *λ*_em_ = 530 nm

C153 anisotropy decay parameters							
[AuNP] (µM)	*α*_1r_	*τ*_1r_ (ps)	*α*_2r_	*τ*_2r_ (ps)	〈*τ*_r_〉 (ps)	*χ* ^2^	*r*_o_
**0.0 mM SDS**							
0.00	0.77 ± 0.01	253 ± 20	0.23 ± 0.01	3815 ± 101	1075 ± 38	1.20	0.37
0.01	0.91 ± 0.02	186 ± 21	0.09 ± 0.01	5056 ± 121	606 ± 30	0.98	0.39
0.02	0.95 ± 0.03	110 ± 67	0.05 ± 0.01	4200 ± 132	315 ± 70	0.98	0.32
0.03	0.97 ± 0.01	85 ± 21	0.03 ± 0.01	3800 ± 119	196 ± 23	1.18	0.31

**0.8 mM SDS**							
0.00	0.56 ± 0.02	1117 ± 101	0.44 ± 0.01	7467 ± 156	3886 ± 125	1.17	0.30
0.01	0.64 ± 0.01	520 ± 98	0.36 ± 0.02	9350 ± 132	3698 ± 110	1.12	0.29
0.02	0.70 ± 0.03	490 ± 87	0.30 ± 0.01	8885 ± 112	2969 ± 94	0.98	0.40
0.03	0.68 ± 0.02	584 ± 101	0.32 ± 0.02	5758 ± 106	2254 ± 102	1.18	0.21

**1.2 mM SDS**							
0.00	0.72 ± 0.01	223 ± 89	0.28 ± 0.01	7007 ± 157	2101 ± 108	1.05	0.14
0.01	0.67 ± 0.03	388 ± 76	0.33 ± 0.03	4507 ± 201	1767 ± 117	1.16	0.18
0.02	0.89 ± 0.02	293 ± 57	0.11 ± 0.02	9932 ± 223	1312 ± 75	1.06	0.33
0.03	0.75 ± 0.01	215 ± 49	0.25 ± 0.01	1332 ± 301	497 ± 112	1.20	0.15

**2.0 mM SDS**							
0.00	0.85 ± 0.02	481 ± 71	0.15 ± 0.03	5348 ± 228	1227 ± 94	1.01	0.09
0.01	0.82 ± 0.01	319 ± 67	0.18 ± 0.02	1527 ± 245	532 ± 99	1.03	0.12
0.02	0.82 ± 0.03	273 ± 57	0.18 ± 0.01	1494 ± 198	496 ± 82	1.01	0.37
0.03	0.99 ± 0.04	131 ± 69	0.01 ± 0.01	5259 ± 158	157 ± 69	0.99	0.03

**Table 4:** Rotational relaxation times (*τ*_1r_, *τ*_2r_) and corresponding weights (*α*_1r_, *α*_2r_), average rotational relaxation time (〈*τ*_r_〉) and limiting anisotropy (*r*_o_) of Rh6G in native EMb bioconjugate (0.0 mM SDS), EMb bioconjugate treated with 0.8 mM of SDS, EMb bioconjugate treated with 1.2 mM of SDS, and EMb bioconjugate treated with 2.0 mM of SDS in the acetate buffer medium at pH 4.5. [EMb bioconjugate] = 10.00 µM, [Rh6G] = 0.5 µM, [AuNPs] = 0 to 0.03 µM. *λ*_ex_ = 510 nm, *λ*_em_ = 560 nm

Rh6G anisotropy decay parameters							
[AuNP] (µM)	*α*_1r_	*τ*_1r_ (ps)	*α*_2r_	*τ*_2r_ (ps)	〈*τ*_r_〉 (ps)	*χ* ^2^	*r*_o_
**0.0 mM SDS**							
0.00	0.89 ± 0.01	182 ± 67	0.11 ± 0.03	6742 ± 117	861 ± 72	1.02	0.13
0.01	0.98 ± 0.01	208 ± 56	0.02 ± 0.01	1634 ± 121	232 ± 57	1.00	0.24
0.02	0.65 ± 0.02	39 ± 12	0.35 ± 0.02	223 ± 98	103 ± 42	1.53	0.15
0.03	0.60 ± 0.03	25 ± 10	0.40 ± 0.03	186 ± 89	89 ± 41	1.44	0.33

**0.8 mM SDS**							
0.00	0.44 ± 0.02	144 ± 43	0.56 ± 0.02	623 ± 67	413 ± 56	0.98	0.29
0.01	0.36 ± 0.02	118 ± 56	0.64 ± 0.03	574 ± 82	409 ± 72	0.98	0.27
0.02	0.26 ± 0.03	130 ± 71	0.74 ± 0.01	466 ± 78	379 ± 76	1.12	0.24
0.03	0.29 ± 0.01	95 ± 12	0.71 ± 0.02	489 ± 34	375 ± 27	1.00	0.28

**1.2 mM SDS**							
0.00	0.14 ± 0.02	1248 ± 104	0.86 ± 0.03	9422 ± 109	8279 ± 108	1.17	0.23
0.01	0.41 ± 0.04	473 ± 98	0.59 ± 0.03	7065 ± 103	4361 ± 100	1.23	0.29
0.02	0.47 ± 0.01	472 ± 79	0.53 ± 0.02	6437 ± 119	3654 ± 100	1.20	0.27
0.03	0.62 ± 0.01	243 ± 65	0.38 ± 0.01	6149 ± 121	2492 ± 86	1.21	0.31

**2.0 mM SDS**							
0.00	0.93 ± 0.02	158 ± 56	0.07 ± 0.01	4102 ± 123	618 ± 60	1.11	0.23
0.01	0.95 ± 0.03	142 ± 36	0.05 ± 0.02	3365 ± 128	472 ± 40	1.09	0.22
0.02	0.96 ± 0.01	121 ± 68	0.04 ± 0.03	2742 ± 114	298 ± 69	1.02	0.31
0.03	0.97 ± 0.02	108 ± 39	0.03 ± 0.01	2118 ± 107	187 ± 41	1.12	0.27

Interestingly, in the amorphous aggregate state, the rotational motions of both dyes become restricted, with slower motions exhibiting greater amplitude than in the native state of the bioconjugates (Tables 3 and 4). Fluorescence lifetime is shortened in the aggregate state due to the dye's closer proximity to AuNPs. However, an increased rotational relaxation time indicates that the microenvironment becomes rigid, even though the protein has a comparatively open structure. It supports the amorphous state of the aggregate. Therefore, the larger *k*_nr_ values for both C153 and Rh6G (Fig. 11 and 12, respectively) are due to a greater energy transfer from the dye to the AuNPs. 〈*τ*_r_〉 values of C153 and Rh6G in the amorphous aggregate state with increasing AuNPs concentration have been plotted in Fig. 13a and b, respectively. Notably, the enhancement in 〈*τ*_r_〉 in this state is substantially higher in the case of C153 than Rh6G (Fig. 13a and b, respectively), suggesting that the interior of the protein is significantly more rigid than the exterior. Results are supported by apparently higher *r*_o_ values for C153 than for Rh6G. Time-resolved fluorescence anisotropy results are in very good agreement with the steady-state fluorescence anisotropy values tabulated in Table S3. The steady-state anisotropy is higher in the aggregate state than in the native bioconjugate state, with a larger value for C-153 than Rh6G.

Conversely, amyloid fibrils exhibit faster anisotropy decay for C153 with a larger amplitude, reflecting weak immobilisation within the fibril core of cross-β secondary structure compared to the amorphous aggregate state. However, Rh6G undergoes substantially slower anisotropy decay than that in the amorphous aggregate state, even with surface-bound motions. For Rh6G, both motions become comparatively restricted with the appearance of a component with a very long rotational relaxation time. In amyloid fibrils, for C153, 〈*τ*_r_〉 values are shorter at all concentrations of AuNPs (Fig. 13a), whereas for Rh6G, 〈*τ*_r_〉 values are significantly longer (Fig. 13b), compared to those in the amorphous aggregate state. Results indicate that the protein's interior is more flexible, while the protein's exterior is more rigid, as compared to the amorphous aggregate state, even when exposed to the outer environment. For the amyloid fibrils, *r*_o_ values are lower for C153, but those are higher for Rh6G, as compared to the amorphous aggregate state. Time-resolved data are well supported by the steady-state fluorescence anisotropy values given in Table S3. While fluorescence anisotropy is reduced for C153, it is significantly enhanced for Rh6G. An important observation of this study is that the cross-β secondary structure implies a rigid outer environment compared to the amorphous aggregate state.

At a higher concentration of SDS, in the partially refolded state, C153 exhibits fast motion as a major component for depolarisation, along with a minor slow component, as found in the native bioconjugates (Table 3). Similar results are obtained for Rh6G as well (Table 4). Fig. 13a and b display the variation of 〈*τ*_r_〉 as a function of AuNPs concentrations for C153 and Rh6G, respectively. Values (Tables 3 and 4) and trends (Fig. 13a and b) are almost identical to those for the native bioconjugate state. Results are in good agreement with the steady-state fluorescence anisotropy data (Table S3), which indicate faster overall motions, similar to those in the native bioconjugate state. Notably, both steady-state and time-resolved fluorescence anisotropy results show that the interior as well as the exterior of both amorphous aggregate state and amyloid fibrils are less flexible than the native bioconjugate and the partially refolded bioconjugate states. These results highlight the distinct motional constraints imposed by disordered *versus* ordered protein aggregates.

**Scheme 2:**
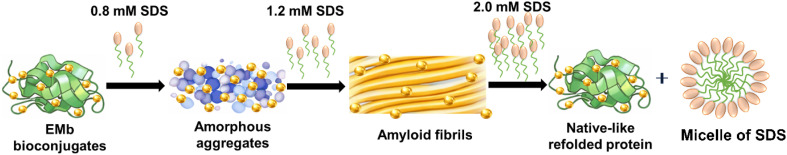
Schematic representation of SDS-induced structural transitions in EMb-AuNP bioconjugates from native conformations to amorphous aggregates, amyloid fibrils, and partially refolded/native-like states.

The overall SDS-induced structural transition pathway of EMb-AuNP bioconjugates is schematically represented in Scheme 2. Fluorescence from intrinsic aromatic amino acid residues, along with C153 and Rh6G as interior- and surface-sensitive probes, respectively, has been employed to demonstrate the microenvironmental heterogeneity and energy-transfer behaviour of different conformational states. Intrinsic fluorescence results show state-dependent quenching predominantly governed by non-radiative decay pathways, with amorphous aggregates exhibiting the highest quenching efficiency due to enhanced structural disorder and greater fluorophore accessibility to AuNPs. An open interior matrix in amorphous aggregates that has promoted maximum energy-transfer efficiency has been revealed by fluorescence-lifetime data on C153. However, the ordered cross-β -sheet architecture of amyloid fibrils has shielded C153 within the fibrillar core, resulting in reduced energy transfer and enhanced excited-state lifetime. Time-resolved fluorescence anisotropy data have indicated that, despite their open structure, amorphous aggregates possessed a comparatively rigid interior microenvironment, while amyloid fibrils exhibited weaker immobilization within the fibrillar core but a relatively rigid exterior environment. Both amorphous aggregates and amyloid fibrils have been found to possess less flexible interior and exterior environments than native bioconjugates. Furthermore, the photophysical behavior of intrinsic and extrinsic fluorophores supported the defibrillation of protein structures at SDS concentrations above the critical micelle concentration. Overall, this study demonstrates the effectiveness of NSET-based photophysical techniques for monitoring protein aggregation, amyloid fibrillation, microenvironmental heterogeneity, and defibrillation processes in bioconjugates. The present approach can be extended to other protein-nanoparticle systems for studying aggregation and amyloid formation.

## 4 Conclusion

A comprehensive investigation of SDS-induced structural transitions in EMb-AuNP bioconjugates has been carried out, which elucidates how nanoparticle proximity governs photophysical behaviour across distinct conformational states. Spectroscopic and microscopic characterization confirmed stable bioconjugate formation and revealed that AuNPs-binding significantly modifies the SDS-driven aggregation pathway, shifting and broadening the fibrillation regime relative to free myoglobin. Turbidity, intrinsic fluorescence, ThT binding, and TEM analyses collectively establish the existence of four well-defined structural states: native, amorphous aggregates, cross-β secondary structure of amyloid fibrils, and partially refolded native-like conformations. Systematic intrinsic fluorescence quenching and fluorescence lifetime measurements of aromatic amino acid residues, and calculations of NSET/FRET parameters, demonstrate that AuNPs-induced fluorescence modulation is dominated by non-radiative decay arising from energy transfer processes. Among the various states of protein, amorphous aggregates exhibit the highest fluorescence quenching efficiency and shortest average fluorescence lifetimes due to pronounced structural disorder and enhanced fluorophore accessibility, whereas amyloid fibrils impose partial protection of aromatic amino acid residues through ordered cross-β architectures. Refolded states of protein above the critical micelle concentration of SDS show partial recovery of photophysical properties, consistent with restored compactness in the protein's structure through defibrillation.

The use of C153 and Rh6G as complementary interior- and surface-sensitive probes, respectively, further reveals spatially selective energy-transfer pathways. While C153 experiences sequestration and enhanced fluorescence lifetime within amyloid cores, Rh6G exhibits maximal quenching and energy transfer in fibrillar states due to persistent surface localization along AuNPs-decorated interfaces. Radiative and non-radiative decay rate constants analysis confirm that changes in energy transfer efficiency arise primarily from structural reorganization rather than intrinsic emissive alterations. The shortest fluorescence lifetime component of C153, with a high amplitude, reveals an open interior matrix of amorphous aggregates of the bioconjugate, allowing the greatest extent of energy transfer from the dye to AuNPs. On the other hand, the ordered cross-β secondary structure of amyloid fibrils shields C153 within the fibril core from its exposure to AuNPs, which enhances the excited state lifetime as a result of limited non-radiative decay due to a longer average donor–acceptor distance. However, a longer rotational relaxation time for C153 in amorphous aggregates indicates a rigid microenvironment within its interior, despite the protein's comparatively open structure. Conversely, C153 exhibits faster fluorescence anisotropy decay with a larger amplitude in amyloid fibrils, reflecting its flexible environment within the fibril core of cross-β secondary structure compared to the amorphous aggregate state, while Rh6G undergoes substantially slower anisotropy decay even with surface-bound motions, revealing the rigid exterior of EMb bioconjugate. Both the interior and exterior environments of amorphous aggregates, as well as amyloid fibrils of protein bioconjugate, are less flexible than the native bioconjugates. The C153 molecule is located farthest from the AuNPs, supporting it as a reporter of the protein's interior. It infers that the protein's aromatic amino acid residues are not buried too deeply inside the protein.

Overall, this work establishes a robust photophysical framework linking protein conformational transitions, aggregation morphology, and nanoparticle-mediated energy transfer. These findings advance our understanding of protein–nanoparticle interactions under aggregation-prone conditions and highlight the utility of combined intrinsic and extrinsic fluorescence probes for resolving nanoscale structural heterogeneity in complex bio-nano assemblies.

## Author contributions

Shalini Dyagala – conceptualization, visualization, methodology, investigation, performing and analysis of experiments, and writing an original draft. Chien-Hsiang Chang – data curation, electron microscopy facility, and resources. Subit K Saha – conceptualization, supervision, visualization, project administration, writing review & editing, resources, funding acquisition.

## Conflicts of interest

There are no conflicts of interest to declare.

## Supplementary Material

RA-016-D6RA02822E-s001

## Data Availability

No software or code has been included as a part of this paper. The data supporting this article have been included as part of the supplementary information (SI). Supplementary information: Experimental details, including materials and methods, and protein sample preparations. Additional methods are available for instruments such as UV-visible, fluorescence, HR-TEM, XPS, PXRD, DLS, zeta potential, turbidity measurements, intrinsic fluorescence spectroscopic measurements, Thioflavin T assay, and Far-UV CD spectroscopy. The method followed to calculate FRET and NSET parameters. Additional figures related to the isoelectric point, CMC, Intrinsic fluorescence spectra of bioconjugates of different systems, Fluorescence spectra showing quenching of fluorescence of C153, Rh6G, and a table related to the % of various elements of secondary structures calculated. See DOI: https://doi.org/10.1039/d6ra02822e.
