# Supplementary material for: Fibrillation/defibrillation of myoglobin decorated with gold nanoparticles probed through nanometal surface energy transfer mechanism
Source: RSC Adv. 2026 Jul 2;16(34):33294–312. doi: 10.1039/d6ra02822e (PMC13326658; doi:10.1039/d6ra02822e)
Supplement: RA-016-D6RA02822E-s001 [file RA-016-D6RA02822E-s001.pdf]

Supplementary Information (SI) for RSC Advances.
This journal is © The Royal Society of Chemistry 2026

Supplementary Information

Fibrillation/Defibrillation of Myoglobin Decorated with Gold Nanoparticles Probed through Nanometal Surface Energy Transfer Mechanism

Shalini Dyagala[a], Chien-Hsiang Chang[b], and Subit Kumar Saha[a*]

[a] *Department of Chemistry, Birla Institute of Technology and Science, Pilani, Hyderabad Campus, Hyderabad, Telangana 500078, India*

[b] *Department of Chemical Engineering, National Cheng Kung University, No. 1, University Rd., East Dist., Tainan 70101, Taiwan*

SECTION S1:

S1. Materials & methods:

S1.1. Materials:

Equine skeletal myoglobin, hydrogen tetrachloroaurate (III), sodium chloride, potassium chloride, sodium phosphate dibasic, potassium phosphate monobasic, Coumarin 153, Rhodamine 6G, trisodium citrate, SDS, Thioflavin T, Anhydrous Sodium Acetate, and glacial acetic acid, all are procured from Sigma Aldrich and used without further purification.

S1.2. Methods

S1.2.1. Protein sample preparation: The stock solution of EMb-AuNP bioconjugates was prepared in a 20 mM phosphate buffer, pH 7.4, and dialyzed overnight in the same buffer. A 0.22 µm PES syringe filter was used to filter dialysis bioconjugates. EMb concentration was calculated using a spectrophotometer at 409 nm with an extinction coefficient of 157,000 M^{-1} cm^{-1}. After equilibration with varying SDS concentrations (0.0 to 5.0 mM) in the bioconjugates (0.2 mg/mL), samples were incubated overnight at room temperature.

S1.2.2. UV-Vis absorption and fluorescence spectral measurements: The absorption spectral studies were recorded using a JASCO (model V-650) UV-Vis spectrophotometer. The fluorescence spectra were recorded using a Horiba Scientific FluoroLog spectrofluorimeter. A quartz cuvette with a Teflon stopper and a 1 cm path length was used in each case. The fluorescence emission spectra and steady-state anisotropy spectra were recorded at a slit width of 3 nm, with a constant scan rate for all measurements. The fluorescence spectra were further corrected to account for instrument sensitivity and to avoid any possible inner-filter effect.

The steady-state fluorescence anisotropy (r) of fluorophores has been measured on the same steady-state spectrofluorimeter using polarizers based on equation S1:

$$r = \frac{[I_{\parallel} - GI_{\perp}]}{[I_{\parallel} + 2GI_{\perp}]} \quad \text{(S1)}$$

where G indicates the correction factor corresponding to detector sensitivity to the emission's polarization detection, and $I_{\parallel}$ and $I_{\perp}$ represent fluorescence intensities polarized parallelly and perpendicularly to the excitation light polarization, respectively. The G-factor is ~ 0.6 for our instrument.

The time-resolved fluorescence and fluorescence anisotropy measurements were performed using a Horiba Delta Flex Modular fluorescence lifetime system using a time-correlated single photon counting (TCSPC) method. The time-resolved fluorescence decays were recorded at a magic angle of 54.7° for the lifetime measurements. Picosecond diode laser sources of 280 nm, 405 nm (NanoLED 405L), and 510 nm (NanoLED 510L) were used as the excitation sources for tryptophan residues, C-153, and Rh6G, respectively. The time-resolved fluorescence measurements were analyzed using EzTime software. The decay profiles were fitted bi-exponentially and tri-exponentially. The fittings were judged by the $\chi 2$ criterion, whose values must lie between 1 and 1.2, and the visual inspection of the residuals of the fitted function to the data. The average excited singlet state lifetime, $\langle\tau_f\rangle$, for a bi-exponential intensity decay was calculated by using equation S2:[15]

$$< \tau_f >= a_1\tau_1 + a_2\tau_2 \quad \text{(S2)}$$

where a_1 and a_2 are relative amplitudes and $a_1 + a_2 = 1$. τ_1 and τ_2 are the lifetimes of the two components, respectively.

The average excited singlet state lifetime, $\langle\tau_f\rangle$, for a tri-exponential intensity decay was calculated by using equation S3:[15]

$$< \tau_f >= a_1\tau_1 + a_2\tau_2 + a_3\tau_3 \quad \text{(S3)}$$

where a_1, a_2 and a_3 are relative amplitudes and $a_1 + a_2 + a_3 = 1$. τ_1, τ_2 and τ_3 are the lifetimes of the three components, respectively.

The time-resolved fluorescence anisotropy, $r(t)$, measurements were carried out on the same TCSPC instrument using polarizers, and $r(t)$ values were determined by equation S4:[15]

$$r(t) = \frac{[I_{\parallel}(t) - GI_{\perp}(t)]}{[I_{\parallel}(t) + 2GI_{\perp}(t)]} \quad \text{(S4)}$$

where G indicates the correction factor corresponding to detector sensitivity to the emission's polarization detection, and $I_{\parallel}(t)$ and $I_{\perp}(t)$ represent fluorescence decays polarized parallelly and perpendicularly to the excitation light polarization, respectively. The G-factor is ~ 0.6 for our instrument. The anisotropy decay function can be described using equation S5:[15]

$$r(t) = r_o \sum_i a_{ir} exp(-{}^{t}/_{\tau_{ir}}) \quad \text{(S5)}$$

Where, r_o symbolizes the limiting anisotropy, which deals with the inherent depolarization of the probe molecule, τ_{ir} stands for rotational relaxation time for rotational motion, i, of the probe molecule, and a_{ir} refers to a corresponding relative amplitude, where $\sum_i a_{ir} = 1$. In the case of the bi-exponential anisotropy decay, the average rotational relaxation time is given by equation S6:[15]

$$< \tau_r >= a_{1r}\tau_{1r} + a_{2r}\tau_{2r} \quad \text{(S6)}$$

where, $a_{1r} + a_{2r} = 1$

k_r and k_{nr} are calculated using Equations S7 and S8, respectively, with the fluorescence quantum yield (f) and average lifetime ($<\tau_f>$) given in the Tables, and are tabulated in the same.[15]

$$k_r = \phi_f / <\tau> \quad \text{(S7)}$$

$$k_{nr} = (1 / <\tau>) - k_r \quad \text{(S8)}$$

S1.2.3. HR-TEM: HR-TEM images were obtained using the A02 Field Emission Cryogenic Transmission Electron Microscope (JEM-2100F Transmission Electron Microscope) of Japan JEOL, model JEM-2100F, with a 200 keV accelerating voltage and a DE-12 Camera System. Images of SDS-treated EMb-AuNP bioconjugate samples were acquired. The effect of SDS on the shape of bioconjugate amorphous aggregates and amyloids was studied by utilizing 10-µL samples containing 0.2 mg/mL EMb in the presence and absence of SDS. The sample was prepared by drop-casting 10 µL of solution on a carbon-coated Cu grid and by drying overnight in a vacuum desiccator. After drying, the samples on the grids were examined using HR-TEM.

S1.2.4. **X-ray photoelectron spectroscopy and Powder X-ray diffraction:** XPS measurements were performed using a Thermo Scientific K-Alpha surface analysis spectrometer with an A1 Kα X-ray source (1486.6 eV). XPS samples were prepared by drop-casting 10.0 µL of the solution onto small pieces of carbon tape and drying under vacuum. These measurements were performed to determine the surface oxidation state of in-situ-synthesized AuNPs in the sample. The C (1s) binding energy at 284.5 eV was used as a reference to compensate for charging effects. The narrow scan for Au was recorded before the

survey scan of all of the elements to avoid any further reduction of unreduced Au (if any) in the solution.

The added $HAuCl_4$. $3H_2O$ in the myoglobin system was characterized by powder XRD (PXRD) to confirm the in-situ synthesis of AuNPs. The powder form of the aqueous system was obtained by freeze-drying. The sample was frozen at −80 °C overnight and then lyophilized to a powder. A dark purple-brown powder obtained after lyophilization was used for XRD characterization. The high-resolution powder X-ray diffraction pattern was obtained using a Rigaku Ultima IV X-ray diffractometer with Cu Kα radiation (λ = 1.5418 Å) at a scan rate of 1° min^{-1} and a step size of 0.01°.

***S1.2.5. Dynamic light scattering (DLS) and Zeta Potential Measurements*:** Hydrodynamic diameter values and zeta potential values were determined using a Zetasizer™ ZEN 3600 instrument (Malvern Instruments Ltd., UK) at 25 °C through the Dynamic Light scattering technique. Prior to measurement, a main stock solution was prepared by effectively filtering PBS buffer through a filter paper with a pore size of 0.22 µm (Durapore, PVDF). The instrument used laser light at 632.8 nm, with a scattering angle of 173°.

S1.2.6. Turbidity measurement: To investigate aggregation, an EMb-AuNP bioconjugate solution (0.2 mg/mL) was incubated overnight at room temperature in the absence and presence of varying concentrations (0.0 to 3.5 mM) of SDS in a 50 mM acetate buffer at pH 4.5 to induce amyloid fibril formation. The turbidity of EMb solution was measured at 650 nm in the absence and presence of SDS using a UV–Vis spectrophotometer.

S1.2.7. Intrinsic fluorescence spectroscopic measurements: The intrinsic fluorescence of EMb-AuNPs bioconjugate samples in the absence and presence of different SDS concentrations was evaluated at room temperature using a 1 cm path length cuvette on a spectrofluorometer.[32,33] These bioconjugate samples (0.2 mg/mL) were excited at 280 nm, and emission spectra were recorded between 300 and 500 nm. Details of the spectrofluorimeter are given above in S1.2.2.

S1.2.8. Thioflavin assay (ThT): The final concentration of the ThT stock solution was determined using absorbance at a molar extinction coefficient of 36,000 M^{-1} cm^{-1}, after preparing a fresh 10 mM ThT stock in MilliQ water and passing it through a 0.45 µm syringe filter. To measure the amyloid-fibril formation in the SDS-treated bioconjugate samples, 10

μM ThT was added to the SDS-treated bioconjugate solution (0.2 mg/mL) and incubated in the dark for a period of 30 min. ThT fluorescence was measured by excitation of SDS-treated EMb samples at 440 nm, and emission spectra were recorded in the spectrofluorimeter from 450 to 650 nm. The excitation and emission slit widths were both set at 3. In addition, Th-T Kinetics tests were performed at a pH of 4.5 to determine SDS-induced amyloid formation in the EMb-AuNP bioconjugate systems.

Scheme S1: Chemical structure of ThT

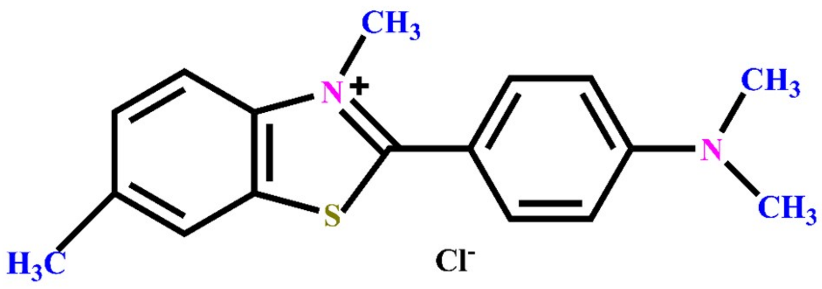

Thioflavin-T (ThT)

S1.2.9. Far-UV CD Spectroscopy: The far–UV circular dichroism (CD) spectra of the required systems were recorded using a Jasco J-1500 CD spectropolarimeter over the wavelength range of 190-260 nm. Here, the cuvette path length was maintained at 0.1 cm. For recording the CD spectrum, a scan speed of 50 nm min^{-1} and a spectral bandwidth of 2.5 nm were used. The buffer spectrum was then subtracted from the recorded spectra of the different EMb systems to correct for background.

S1.3: FRET and NSET Methods

S1.3.1: FRET Method

FRET is a radiationless energy-transfer process between the donor and the acceptor. Energy is transferred from a donor to an acceptor through dipole-dipole interactions, without photon emission or reabsorption. Increasing the acceptor concentration quenches the donor's fluorescence. According to Förster's theory, the FRET efficiency depends on the following factors: (i) the extent of overlap between the donor's fluorescence and the acceptor's absorption, (ii) the distance between the donor and the acceptor (acceptable range is ~2–9 nm) and (iii) orientation between the donor's and the acceptor's transition dipoles. Here, the dye

acts as a donor in energy transfer to the acceptor (AuNPs). The distance at which 50% energy transfer takes place between the donor and the acceptor is called Förster's distance (R_o), which is calculated using Equation S9 given below:[15]

$$R_O = 0.211 \times \left[\kappa^2 \eta^{-4} Q_D J(\lambda)\right]^{1/6} \qquad \text{(S9)}$$

where $J(\lambda)$ represents the overlap integral, and it's calculated by Equation S10, which measures the extent of overlap between the emission and absorption spectra of the donor and acceptor moieties, respectively. Its unit is $M^{-1}cm^{-1}(nm)$.[15]

$$J(\lambda) = \frac{\int_0^\infty F_D(\lambda)\varepsilon_A(\lambda)\lambda^4 d\lambda}{\int_0^\infty F_D(\lambda) d\lambda} \qquad \text{(S10)}$$

The molar extinction coefficient of the acceptor and the corrected fluorescence intensity of the donor at a given wavelength (λ) are denoted as $\varepsilon_A(\lambda)$ and $F_D(\lambda)$, respectively. κ^2 symbolizes the relative orientations corresponding to the transition dipoles of the donor and the acceptor moieties residing in space. Its value is found to be 2/3 by averaging the donor and acceptor dynamic motions. The refractive index of the PBS buffer medium is given as η (with a value of 1.5), while Q_D refers to the quantum yield of the donor in the absence of the acceptor. The Ro values are calculated using a MATLAB program. The energy transfer efficiency (E_T) has been determined by the following Equation S11.[15]

$$E_T = 1 - \frac{F_{DA}}{F_D} \qquad \text{(S11)}$$

where F_D and F_{DA} represent fluorescence intensities of the dye in the absence and in the presence of *in-situ* synthesized AuNPs. The average distance (r) between the dye and *in-situ* synthesized AuNPs has been determined using the values of R_o and E_T using Equation S12:[15]

$$E_T = \frac{R_0^{\,6}}{R_0^{\,6} + r^6} \qquad \text{(S12)}$$

S1.3.2: NSET Method

In the case of a noble metal surface, the rate of surface energy transfer (k_{NSET}) depends on the factor $1/d^4$. k_{NSET} can be written in a simplified version as Equation S13:[15]

$$k_{NSET} = \left(1/\tau_D\right)\left(d_0/d\right)^4 \tag{S13}$$

where τ_D is the donor's lifetime in the absence of NPs and d_o is the distance at which a dye displays equal probabilities for energy transfer and spontaneous emission. The value of d_o is calculated using the Persson model[15] as Equation S14:[15]

$$d_o = \left(0.225c^3\Phi_d/\omega_D{}^2\omega_F k_F\right)^{1/4} \tag{S14}$$

where c refers to the speed of light, Φ_d is the quantum yield of the donor, the frequency of the donor electronic transition is ω_D (taken as 6.8×10^{15} s^{-1}), the Fermi frequency is ω_F (taken as 8.4×10^{15} s^{-1}), and the Fermi wave vector is k_F of the metal. The efficiency of surface energy transfer can be written as Equation S15:[15]

$$E_T = \frac{1}{1 + (\frac{d}{d_o})^4} \tag{S15}$$

The E_T can be easily calculated using Equation S11. Once the values of d_o and E_T are calculated, the value of d can be estimated.

In all experimental measurements, the temperature was uniformly maintained at 298.15 ± 1K.

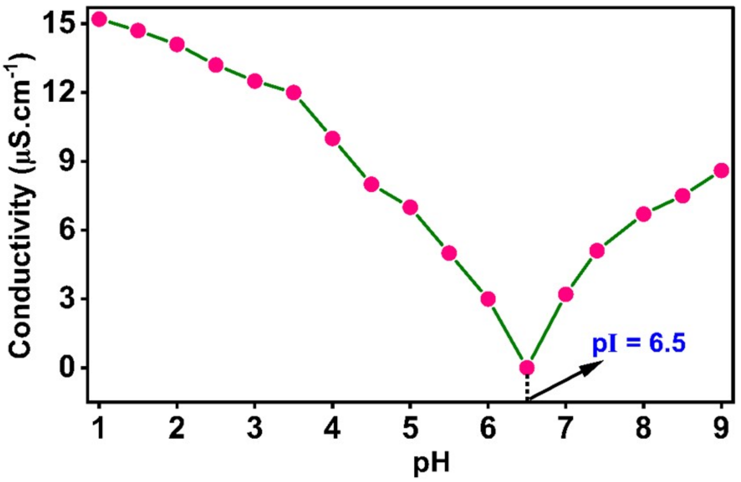

Figure S1: Conductivity of the solution of EMb bioconjugates at different pH. pI = Isoelectric point of EMb bioconjugate.

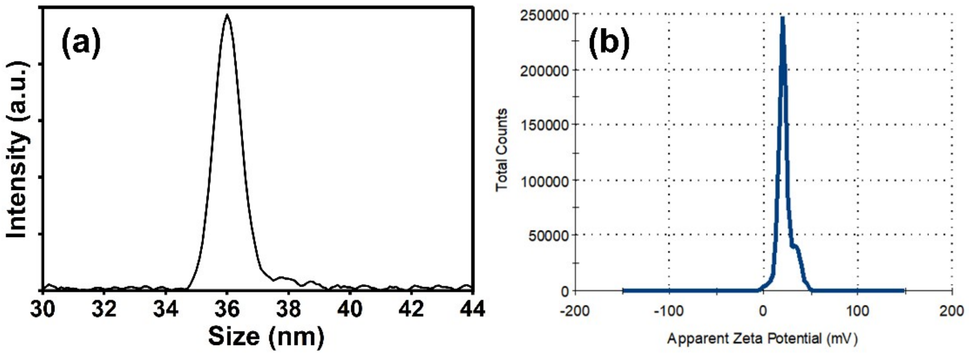

Figure S2: (a) Dynamic light scattering (DLS) and (b) Zeta potential distribution profile of EMb-AuNP bioconjugates.

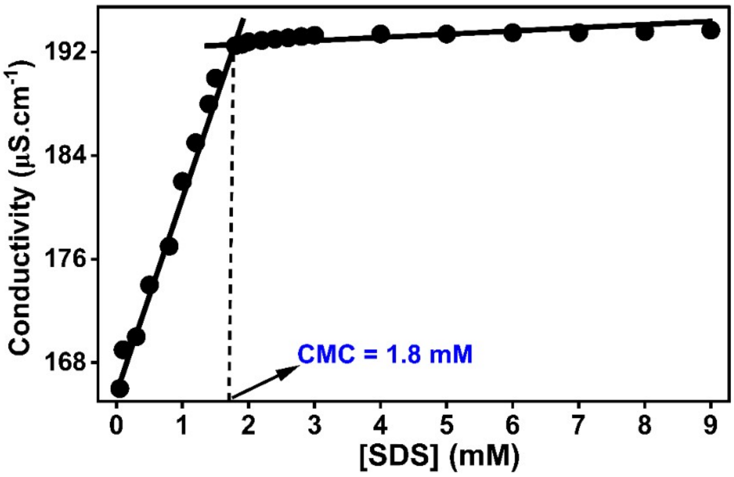

Figure S3: Conductivity of solutions of EMb bioconjugates as a function of increasing SDS concentrations in the acetate buffer at pH 4.5 ([SDS] = 0.0 to 9.0 mM).

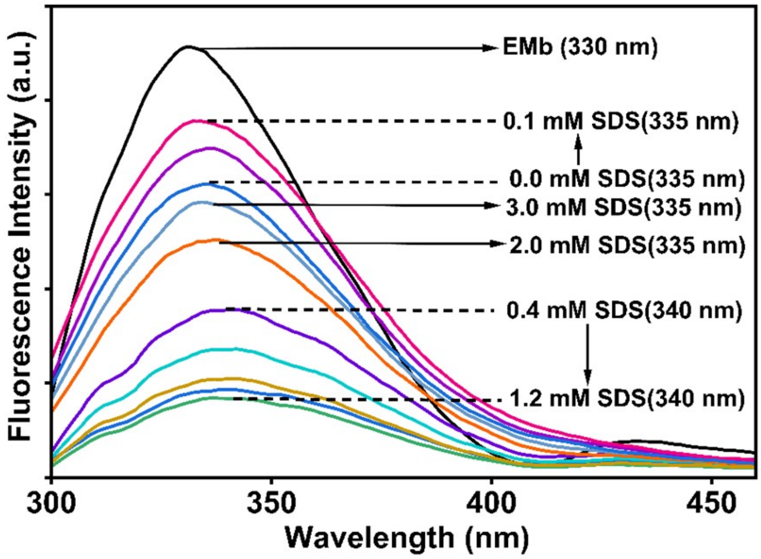

Figure S4: Intrinsic fluorescence spectra of the EMb-AuNP bioconjugates treated with different SDS concentrations in the acetate buffer at pH 4.5. λ_{ex} = 280 nm. [EMb] = [EMb bioconjugate] = 10.00 μM, [AuNPs] = 0.0124 μM, and [SDS] = 0.0 to 3.0 mM. EMb is native

**System**	***α*-Helix**	***β*-Sheet**		***β*-turn**	**Random coil**
		**Anti-parallel**	**Parallel**		
EMb	60.0	6.5	3.5	12.0	18.0
EMb Bioconjugates	57.0	7.2	3.3	13.5	19.0
0.8 mM SDS	1.3	22.3	6.6	22.2	47.6
1.2 mM SDS	5.5	36.6	12.6	14.6	30.7
2.0 mM SDS	30.9	15.9	6.7	29.2	17.3

myoglobin without AuNPs.

Table S1: % of various elements of secondary structures of EMb bioconjugates in different conditions.

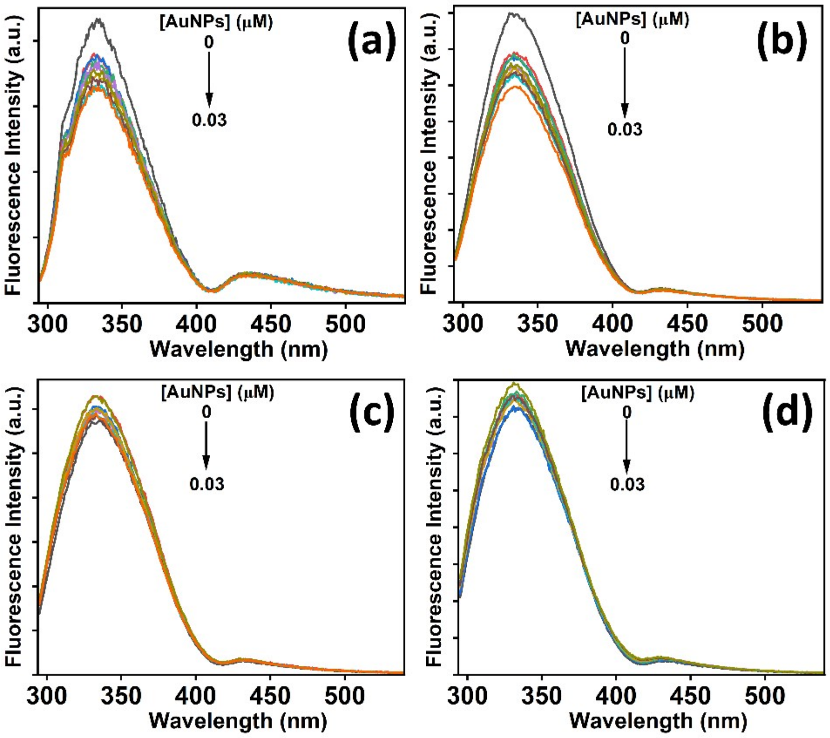

Figure S5: Intrinsic fluorescence quenching plots for (a) native EMb bioconjugate, (b) EMb bioconjugate treated by 0.8 mM of SDS, (c) EMb bioconjugate treated by 1.2 mM of SDS, and (d) EMb bioconjugate treated by 2.0 mM of SDS with increasing concentrations of AuNPs in the acetate buffer at pH 4.5. λ_{ex} = 280 nm. The range of [AuNPs] is from 0.00 to 0.03 μM.

Table S2: Excited singlet state lifetimes (τ_1, τ_2, τ_3) and corresponding weights (α_1, α_2, α_3), average lifetime ($\langle\tau_f\rangle$), fluorescence quantum yield (ϕ_f), radiative (k_r) and non-radiative (k_{nr}) rate constants of intrinsic fluorophores in native EMb bioconjugate, EMb bioconjugate treated with 0.8 mM, 1.2 mM, and 2.0 mM of SDS in the acetate buffer at pH 4.5. λ_{ex} = 280 nm, λ_{em} = 330 nm. [EMb bioconjugate] = 10.00 μM, [AuNPs] = 0.00 to 0.03 μM.

[AuNP] (μM)	α_1	τ_1 (ps)	α_2	τ_2 (ps)	α_3	τ_3 (ps)	$\langle\tau_f\rangle$ (ps)	χ^2	ϕ_f	k_r (× 10^6 s^{-1})	k_{nr} (× 10^8 s^{-1})
**0.0 mM SDS**											
0.00	0.14±0.02	2615±121	0.58±0.02	1652±132	0.28±0.04	4962±127	2714±129	1.15	0.0036	1.33	3.67
0.01	0.31±0.01	2434±131	0.44±0.02	1495±157	0.25±0.02	5065±119	2679±139	1.13	0.0034	1.27	3.72
0.02	0.29±0.02	2582±126	0.48±0.02	1447±154	0.23±0.02	5113±156	2629±146	1.07	0.0032	1.22	3.79
0.03	0.27±0.03	1647±189	0.62±0.01	173±143	0.11±0.01	4669±123	1055±153	1.13	0.0040	3.79	9.44
**0.8 mM SDS**											
0.00	0.07±0.04	825±159	0.02±0.01	2769±181	0.91±0.04	161±20	257±32	1.05	0.0006	2.33	38.89
0.01	0.06±0.01	937±164	0.02±0.01	2876±175	0.92±0.03	139±89	242±95	1.05	0.0005	2.07	41.30
0.02	0.05±0.01	881±132	0.02±0.01	2802±146	0.93±0.03	111±76	199±80	1.15	0.0004	2.01	50.23
0.03	0.04±0.02	1060±123	0.01±0.01	2889±139	0.95±0.03	113±54	179±57	1.07	0.0003	1.68	55.85
**1.2 mM SDS**											
0.00	0.74±0.03	1287±139	0.10±0.01	2984±116	0.16±0.02	4391±121	1949±133	1.14	0.0018	0.92	5.12
0.01	0.73±0.02	1235±128	0.06±0.02	2173±143	0.21±0.03	4274±134	1929±130	1.03	0.0016	0.83	5.17
0.02	0.69±0.03	439±26	0.21±0.02	1950±186	0.10±0.01	4483±189	1154±75	1.14	0.0017	1.47	8.65
0.03	0.76±0.02	391±78	0.17±0.02	1818±276	0.07±0.01	4441±176	906±118	1.16	0.0015	1.66	11.02
**2.0 mM SDS**											
0.00	0.04±0.02	2178±147	0.75±0.02	1118±111	0.21±0.03	4269±197	1834±130	1.17	0.0025	1.36	5.44

0.01	0.03±0.01	2377 ±178	0.75±0.03	1112 ±147	0.22±0.02	4168 ±198	1820 ±159	1.17	0.0026	1.43	5.48
0.02	0.08±0.04	2541 ±189	0.73±0.04	1039 ±177	0.19±0.02	4371 ±184	1792 ±179	1.18	0.0024	1.34	5.57
0.03	0.07±0.02	2411 ±112	0.73±0.01	1039 ±109	0.20±0.01	4133 ±188	1744 ±125	1.16	0.0028	1.61	5.72

Table S3: Steady state fluorescence anisotropy of intrinsic fluorophores in native EMb bioconjugate, EMb bioconjugate treated with 0.8 mM, 1.2 mM, and 2.0 mM of SDS in the absence of dyes, in the presence of C153 and Rh6G dyes, with increasing [AuNPs] from 0.00 to 0.03 μM in the acetate buffer at pH 4.5. [EMb bioconjugate] = 10.00 μM.

**Steady-state fluorescence anisotropy**			
**[AuNP] (μM)**	**Intrinsic fluorophore** λ_{ex} = 280 nm, λ_{em} = 330 nm	**C 153** λ_{ex} = 409 nm, λ_{em} = 535 nm	**Rh6G** λ_{ex} = 510 nm, λ_{em} = 560 nm
**0.0 mM SDS**			
0.00	0.085±0.009	0.018±0.002	0.011±0.002
0.01	0.068±0.004	0.016±0.001	0.010±0.001
0.02	0.059±0.003	0.015±0.002	0.009±0.002
0.03	0.043±0.002	0.012±0.002	0.006±0.001
**0.8 mM SDS**			
0.00	0.062±0.003	0.158±0.009	0.059±0.002
0.01	0.055±0.002	0.131±0.006	0.056±0.003
0.02	0.054±0.004	0.123±0.005	0.053±0.004
0.03	0.034±0.003	0.109±0.007	0.048±0.003
**1.2 mM SDS**			
0.00	0.064±0.003	0.095±0.005	0.225±0.010
0.01	0.058±0.002	0.094±0.006	0.211±0.007
0.02	0.056±0.003	0.093±0.003	0.182±0.009
0.03	0.042±0.003	0.086±0.002	0.168±0.006
**2.0 mM SDS**			
0.00	0.068±0.004	0.032±0.001	0.017±0.001
0.01	0.059±0.003	0.031±0.002	0.012±0.002
0.02	0.058±0.001	0.029±0.002	0.010±0.002
0.03	0.039±0.002	0.026±0.002	0.008±0.001

Table S4: FRET and NSET parameters for native EMb bioconjugate (0.0 mM SDS), EMb bioconjugate treated with 0.8 mM, 1.2 mM, and 2.0 mM of SDS with increasing AuNPs concentration in the acetate buffer at pH 4.5. [EMb bioconjugate] = 10.00 µM, [AuNPs] = 0.003 to 0.030 µM.

[AuNP] (µM)	J ($M^{-1}cm^{-1}(nm)^4$)	R_o (nm)	r (nm)	d (nm)	d_o (nm)	E_T
**0.0 mM SDS**						
0.003	1.27E+20	13.65	22.56±1.56	2.87±0.36	1.37	0.050±0.003
0.010	1.40E+20	13.50	19.96±1.17	2.49±0.30	1.39	0.090±0.010
0.020	1.40E+20	13.42	19.17±1.18	2.34±0.32	1.37	0.100±0.020
0.030	1.31E+20	13.34	18.86±1.21	2.32±0.36	1.38	0.110±0.020
**0.8 mM SDS**						
0.003	1.20E+20	13.76	18.37±1.36	1.58±0.21	1.18	0.15±0.02
0.010	1.18E+20	13.69	17.83±1.12	1.52±0.24	1.17	0.17±0.03
0.020	1.11E+20	13.43	16.27±1.08	1.39±0.19	1.05	0.24±0.04
0.030	1.20E+20	13.32	15.72±1.11	1.31±0.18	1.11	0.27±0.04
**1.2 mM SDS**						
0.003	1.13E+20	13.55	18.80±1.15	1.68±0.25	1.01	0.12±0.02
0.010	1.08E+20	13.43	18.14±1.13	1.58±0.22	1.00	0.14±0.02
0.020	1.05E+20	13.38	17.10±1.16	1.42±0.18	0.99	0.20±0.03
0.030	9.69E+19	13.38	15.96±1.09	1.37±0.18	1.02	0.24±0.03
**2.0 mM SDS**						
0.003	1.49E+20	13.68	22.63±1.68	2.96±0.30	1.39	0.050±0.003
0.010	1.24E+20	13.42	19.91±1.16	2.56±0.37	1.41	0.080±0.010
0.020	1.42E+20	13.34	19.62±1.19	2.41±0.36	1.35	0.090±0.010
0.030	1.29E+20	13.40	19.31±1.16	2.42±0.39	1.39	0.100±0.020

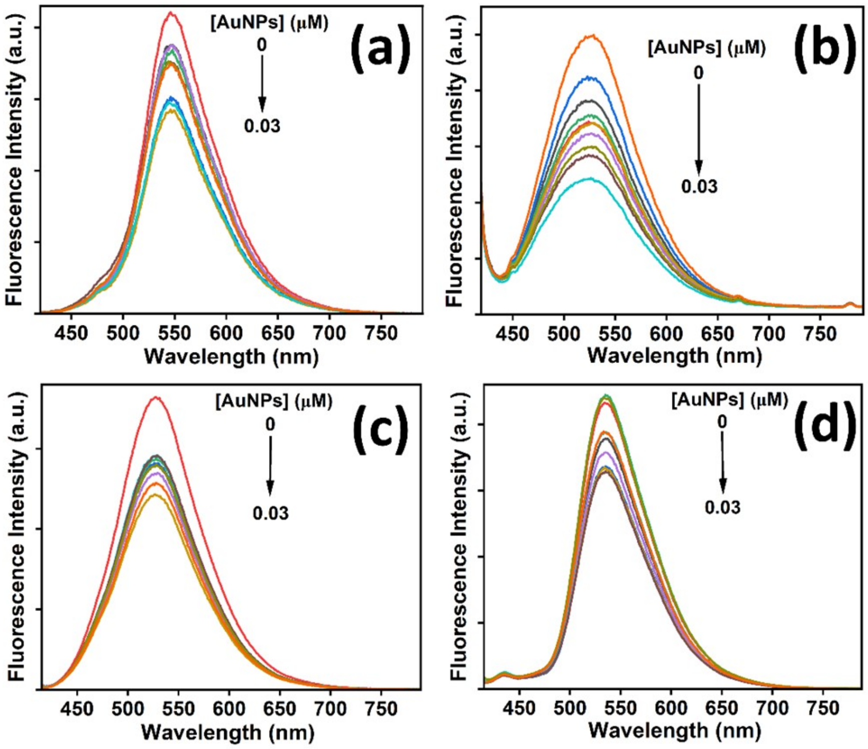

Figure S6: Fluorescence spectra showing quenching of fluorescence of C-153 in (a) native EMb bioconjugate, (b) EMb bioconjugate treated by 0.8 mM of SDS, (c) EMb bioconjugate treated by 1.2 mM of SDS, and (d) EMb bioconjugate treated by 2.0 mM of SDS with increasing concentrations of AuNPs in the acetate buffer at pH 4.5. λ_{ex} = 409 nm. The range of [AuNPs] is from 0.00 to 0.03 μM.

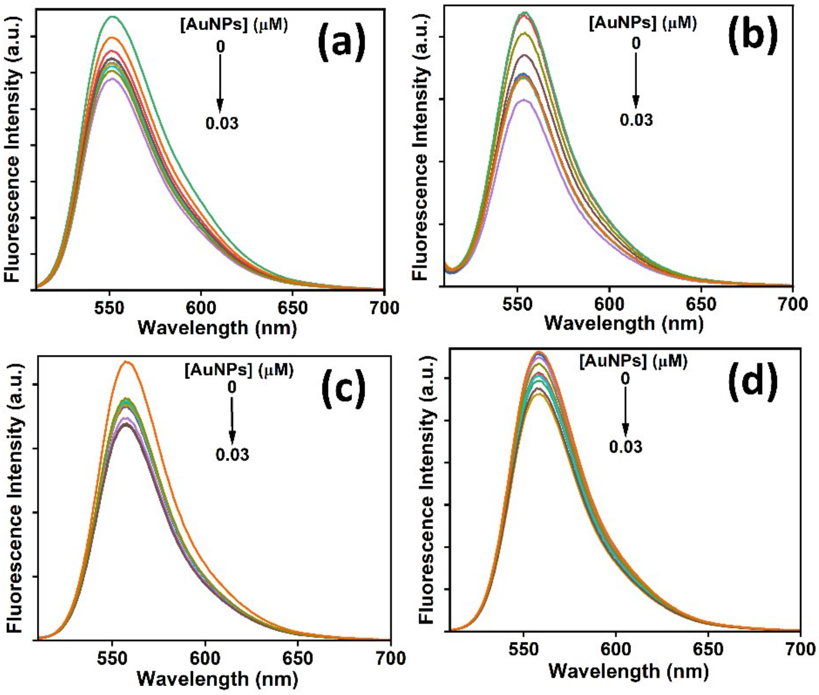

Figure S7: Fluorescence spectra showing quenching of fluorescence of Rh6G in (a) native EMb bioconjugate, (b) EMb bioconjugate treated by 0.8 mM of SDS, (c) EMb bioconjugate treated by 1.2 mM of SDS, and (d) EMb bioconjugate treated by 2 mM of SDS with increasing concentrations of AuNPs in the acetate buffer at pH 4.5. λ_{ex} = 510 nm. The range of [AuNPs] is from 0.00 to 0.03 μM.

Table S5: FRET and NSET parameters for C153 calculated for native EMb (0.0 mM SDS), EMb treated with 0.8 mM of SDS, EMb treated with 1.2 mM of SDS, and EMb treated with 2.0 mM of SDS with increasing AuNPs concentration after loading with C153 in the acetate buffer at pH 4.5. [EMb bioconjugate] = 10.00 μM, [C153] = 0.5 μM, [AuNPs] = 0.00 to 0.03 μM.

**C153**						
**[AuNP] (μM)**	***J*** **($M^{-1}cm^{-1}(nm)^4$)**	***R*₀ (nm)**	***r*** **(nm)**	***d*** **(nm)**	***d*₀ (nm)**	***E*T**
**0.0 mM SDS**						
0.003	1.14E+21	47.68	72.06±3.54	10.11±0.99	5.44	0.08±0.01
0.010	8.05E+20	45.52	62.74±2.31	8.95±0.83	5.53	0.13±0.03
0.020	7.38E+20	43.72	58.29±1.99	8.15±0.83	5.29	0.15±0.02
0.030	7.55E+20	44.50	57.80±2.34	8.09±0.69	5.43	0.17±0.03
**0.8 mM SDS**						
0.003	1.44E+21	26.95	33.90±2.18	3.07±0.38	2.18	0.20±0.03
0.020	1.40E+21	27.18	33.13±2.06	2.99±0.31	2.22	0.23±0.03
0.030	2.12E+21	27.39	31.12±2.11	2.81±0.42	2.32	0.32±0.04
0.030	1.30E+21	26.08	29.32±2.56	2.54±0.31	2.13	0.33±0.04
**1.2 mM SDS**						
0.003	2.25E+21	42.88	58.79±2.16	3.69±0.71	2.29	0.13±0.02
0.010	1.57E+22	39.25	52.12±1.98	3.23±0.35	2.11	0.15±0.01
0.020	1.24E+21	38.34	47.92±2.28	3.06±0.29	2.21	0.21±0.03
0.030	1.52E+22	37.98	46.47±2.37	2.74±0.33	2.02	0.23±0.03
**2.0 mM SDS**						
0.003	1.54E+22	40.24	86.98±3.88	11.66±0.87	3.70	0.010±0.002
0.010	1.95E+21	48.26	81.50±3.22	10.63±0.99	4.84	0.040±0.003
0.020	1.79E+21	47.87	71.32±2.67	8.89±0.99	4.89	0.080±0.004
0.030	1.89E+21	47.25	62.92±2.34	8.37±0.69	5.44	0.150±0.030

Table S6: FRET and NSET parameters for Rh6G calculated for Native EMb (0.0 mM SDS), EMb treated with 0.8 mM of SDS, EMb treated with 1.2 mM of SDS, and EMb treated with 2.0 mM of SDS with increasing AuNPs concentration after loading with Rh6G in the acetate buffer at pH 4.5. [EMb bioconjugate] = 10.00 μM, [Rh6G] = 0.5 μM [AuNPs] = 0.00 to 0.03 μM.

**Rh6G**						
**[AuNP] (μM)**	***J*** **($M^{-1}cm^{-1}(nm)^4$)**	**R_o (nm)**	***r*** **(nm)**	***d*** **(nm)**	**d_o (nm)**	**E_T**
**0.0 mM SDS**						
0.003	1.48E+21	18.16	25.94 ±1.51	2.04±0.24	1.20	0.10±0.03
0.010	8.51E+20	16.46	22.86±1.81	1.94±0.31	1.19	0.12±0.05
0.020	1.19E+21	17.27	22.51±1.66	1.78±0.28	1.18	0.16±0.03
0.030	1.08E+21	17.06	22.47±1.72	1.74±0.27	1.17	0.17±0.02
**0.8 mM SDS**						
0.003	6.87E+21	20.11	19.98±1.11	1.14±0.05	0.90	0.28±0.01
0.010	1.14E+21	16.50	18.59±1.32	0.95±0.22	0.80	0.33±0.03
0.020	8.95E+21	15.97	17.71±1.10	0.91±0.12	0.78	0.35±0.04
0.030	5.14E+21	11.29	14.18±1.00	0.71±0.14	0.72	0.51±0.04
**1.2 mM SDS**						
0.003	6.16E+20	14.21	12.94±0.83	0.78±0.21	0.89	0.63±0.06
0.010	5.09E+20	13.92	12.16±1.02	0.67±0.27	0.81	0.68±0.05
0.020	8.08E+20	14.90	11.99±0.88	0.43±0.11	0.60	0.79±0.03
0.030	1.18E+21	15.95	11.11±0.55	0.21±0.01	0.42	0.87±0.02
**2.0 mM SDS**						
0.003	5.90E+20	24.67	48.93±2.12	6.67±0.51	2.39	0.020±0.003
0.010	1.14E+21	27.92	41.70±2.54	4.45±0.49	2.44	0.070±0.003
0.020	6.62E+20	24.69	37.71±2.34	4.38±0.44	2.32	0.080±0.002
0.030	7.13E+20	25.15	36.67±2.28	4.12±0.61	2.34	0.090±0.003
